# Comprehensive Analysis of Phenolic Compounds in *Solanum glaucophyllum* Desf.

**DOI:** 10.1021/acs.jafc.4c11264

**Published:** 2025-03-19

**Authors:** Thomas Heymann, Sabrina Autzen, Marcus A. Glomb

**Affiliations:** † Martin-Luther-University Halle-Wittenberg, Institute of Chemistry − Food Chemistry, Kurt-Mothes-Str. 2, Halle/Saale D-06120, Germany; ‡ Herbonis Animal Health GmbH, Rheinstrasse 30, Augst CH-4302, Switzerland

**Keywords:** solanum glaucophyllum desf., quercetin derivatives, glucaric acids, quinic acids, secondary plant substances, permethylation

## Abstract

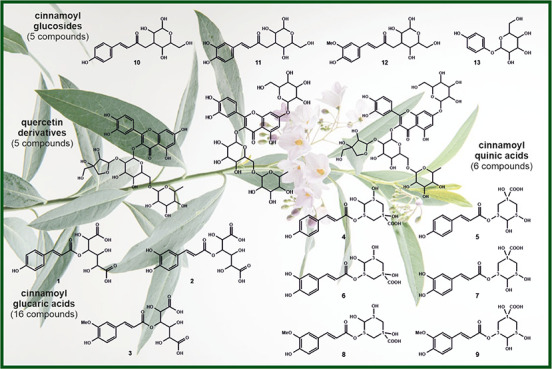

*Solanum glaucophyllum* Desf. (SG) has been well studied due to the presence of 1,25(OH)_2_D_3_ glycosides, but little is known about the composition and presence of other phenolic glycosides that are also part of the plant’s composition. In fact, only 8 phenolic ingredients have been described in the literature; thus, the current study aimed to comprehensively extend the phenolic composition. Aqueous leaf extracts were separated by reversed-phase chromatography or, after permethylation, on normal phase chromatography. Two novel quercetin derivatives were isolated, including 7-*O*-β-glucosyl-α-apiosyl rutin, which was never reported in the literature before. In total, five quercetin glycosides containing one to four sugar units were determined quantitatively for the first time in SG, reaching a total 4.16% dry matter. Additionally, arbutin and 27 cinnamoyl derivatives were identified and quantitated via LC-MS/MS, totaling 0.57% and 2.15% dry matter of leaves, respectively. The quantitative results were based on independent syntheses of 1-*O*-and 3-*O*-caffeoyl- and 3-*O*-feruloyl glucoside, isolation of 3/4-*O*-caffeoyl- and 3/4-*O*-*p*-coumaroyl glucaric acid from SG leaves, and the use of authentic reference material (3- and 5-*O*-cinnamoyl quinic acids). All of the isolated and synthesized substances were unequivocally verified by HR-MS and NMR spectroscopy.

## Introduction

*Solanum glaucophyllum* Desf. (SG) became of interest in the early 20th century due to cattle showing symptoms of enzootic calcinosis after excessive consumption of the plant.^1^ Since this observation, almost all publications on SG focused on the calcium homeostasis-modulating bioactivity, e.g., leading to increased serum levels of calcium and phosphate.^2,3^ The presence of 1,25-(OH)_2_-cholecalciferol (1,25(OH)_2_D_3_) glycosides was first indirectly concluded because aqueous extracts of the plant, especially, had effects on animals.^4,5^ Biosynthetic formation of 1,25(OH)_2_D_3_ was subsequently proven in numerous publications after alkaline or enzymatic hydrolysis, with mostly β-glucosidases.^4,6^ However, up to now, only two 1,25(OH)_2_D_3_ glycosides were isolated from SG and the structures were completely elucidated. This resulted in identification of 1-*O*-1,25(OH)_2_D_3_ glucoside and 1,3-*O*-1,25(OH)_2_D_3_ diglucoside.^7,8^ Vidal et al. suggested a more complex situation and proposed linkages of 2, 4, or 8 glucose units with terminal fructose in all cases, based on hydrolyzed samples.^9^ In detail, Vidal’s approach was not based on native isolated substances, which represents a general problem in the pertinent literature concerning the phytochemical investigation of SG extracts.

Due to the major focus on vitamin D derivatives and animal’s health, other publications of metabolites in SG are scarce. To our knowledge, only Rappaportt et al. determined a total of eight phenolic substances isolated from SG.^10^ They reported on six flavon-3-ol derivatives with one to three sugar units connected to quercetin, kaempferol, and isorhamnetin. Arbutin and methylarbutin were also demonstrated, representing monoglucosides of *p*-hydroquinone and its methoxy derivative, respectively.^10^ In general, the phenolic patterns of other *Solanaceae* species also provide only incomplete pictures. Most publications focus on the toxic steroidal alkaloids or on saponins.^11,12^ Steroidal alkaloids are prominent in the *Solanum* family. Most of the well-documented metabolites belong to C_27_-cholestanes characterized by the common ABCD steroid backbone, leading to three main types: spirosolane, solanidane, and verazine. These steroids typically occur in nature as glycosides with high structural diversity, while galactose (gal), glucose (glc), xylose (xyl), arabinose (ara), and rhamnose (rha) are most common, forming di-, tri-, and tetra-saccharides such as solatriose (gal, glc, and rha), chacotriose (glc, rha, and rha), lycotetraose (gal, glc, xyl, and glc), or commertetraose (gal, glc, glc, and glc).^13^ However, there is no publication for steroidal alkaloids in SG, underlining the lack of metabolite analyses. The same accounts for steroidal saponins, which are also characteristic for *Solanum* species. Over 130 compounds are known, while 32 were identified from *Solanum torvum*.^14^ As a common feature, a perhydrocyclopentenophenanthrene moiety (rings A–D) with an acyclic side chain forms the aglycon backbone. Glc, gal, xyl, ara, and rha are typical constituents of the hydrophilic conjugate, with one to five monosaccharides linked linearly or with one or more branched chains.^15^ Again, nothing was reported for SG. In addition, most phytochemical approaches in current literature are based on high-resolution mass spectrometry, leading to numerous deduced structures; i.e., the resulting phenol profile is often solely based on calculated molecular formulas.^16−18^ This also leads to different isomers with the same nominal mass; however, an unequivocal structural elucidation and differentiation are missing. Some works succeeded in the quantitation of specific compounds by the use of commercially available authentic reference material. In addition, especially the edible parts of selected *Solanaceae* species—such as tomatoes, potatoes, eggplants, or various berries—were in focus with an almost exclusive view on chlorogenic acids and quercetin derivatives.^16−19^

Thus, the current study significantly extended the phenolic profile of SG leaves to 33 different structures on both a qualitative and quantitative basis, comprising glucosides and glucaric acids of quercetin and cinnamic acids. Reversed-phase chromatography was combined with normal-phase chromatography after permethylation. While 29 structures were novel for SG, this is the first report on 7-*O*-β-glucosyl-α-apiosyl rutin.

## Material and Methods

### Chemicals

Chemicals with the highest quality available were obtained from Sigma-Aldrich (Germany), VWR (Germany), TCI (Japan), and Carl Roth AG (Germany), unless otherwise indicated.

### Material

Plant material was provided by Herbonis Animal Health GmbH (Augst, Switzerland). All experiments were performed on Hervit 153 – a protected variety of *Solanum glaucophyllum* Desf. (grant of community plant variety right by CPVO (Community Plant Variety Office) decision No. EU 50806 of 17 December 2018, taken in accordance with Council Regulation (EC) No. 2100/94).

### Extraction of SG

Dried and finely ground leaf material of *Solanum glaucophyllum* Desf. was extracted with a mixture of acetone/water (7/3, *v*/*v*, 30 mL per 5.0 g of plant material) for 30 min by ultrasonication at room temperature. The crude extract was centrifuged (4500 rpm, 5 min), and the supernatant was filtered through a paper filter. The remaining residue was extracted as described for 2 more times. Acetone was then removed from the combined liquid phases on a rotary evaporator at 40 °C. The resulting aqueous suspension was freeze-dried to result in a dark-green to brown crude extract with a yield of about 26% with respect to the dry plant material.

### Hydrolysis of Crude Extract

About 5 mg of above crude extract was reacted in 1.0 mL of 2 N hydrochloric acid at 80 °C in a drying oven (FD 53, Binder, Tuttlingen, Germany) for total acidic hydrolysis. An aliquot of 200 μL was mixed with 200 μL of dimethylformamide prior to injection on the HPLC-DAD system as described below. Retention times of relevant flavon-3-ols, such as quercetin, kaempferol, and isorhamnetin, were determined by the use of authentic reference material, allowing the assignment of released aglycones.

### Pre-Fractionation on RP-18 Column

**Figure 1:**
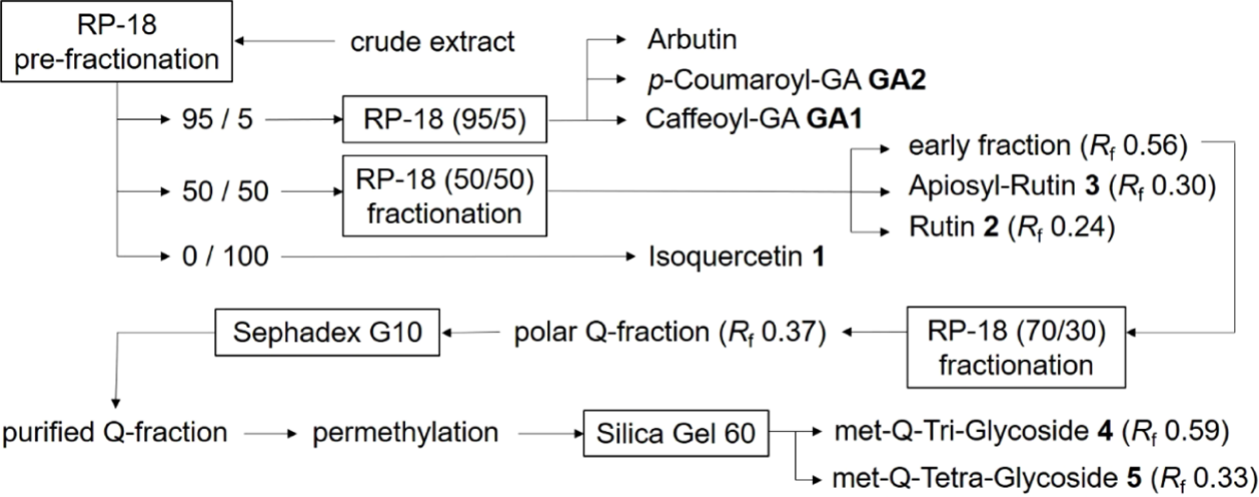
Schematic overview for isolation of seven pure substances from SG leaves starting from a crude extract using different chromatographic systems and derivatization; GA: glucaric acids; Q: quercetin; RP-eluents: *v*/*v* H_2_O/MeOH; met: methylated.

The first fractionation of the crude extract was performed by atmospheric pressure RP-18 chromatography (Figure 1). LiChroprep RP-18 (40–63 μm) was used as the stationary phase, packed in a glass column (30 × 300 mm), with first a mixture of water/methanol (95/5, *v*/*v*) as the mobile phase to elute very polar substances. The obtained fraction was then freeze-dried to result in a light-yellow, amorphous residue (yield: 11% with respect to dry plant material). The mobile phase was then changed to water/methanol (50/50, *v*/*v*) to elute more lipophilic substances. The brown fraction was evaporated to remove methanol to allow freeze-drying afterward. The obtained middle-polar fraction was a deep-brown powder with a yield of about 11%, referred to the dry plant material (42% of the crude extract). This fraction mainly contained quercetin derivatives, as demonstrated by HPLC-DAD and HPLC-MS.

### Fractionation of the Polar Substances

About 400 mg of the above polar fraction (water/methanol, 95/5) was injected to a preparative low-pressure RP-18 system. The mobile phase consisted of water/methanol (95/5, *v*/*v*) containing formic acid (0.8 mL L^–1^) and was used under isocratic conditions. The flow rate was 5.0 mL min^–^^1^ (pump: Waters 510, Millipore, Milford, USA) running through a reversed-phase column (310–25, LiChroprep RP-18 (40–63 μm), Merck, Darmstadt, Germany). One fraction was collected per minute for at least 140 min (fraction collector: Labocol Vario 4000, Labomatic, Allschwil, Switzerland). Eluting substances were detected by a UV detector (Gynkotec SP-6, Germering, Germany) set at 300 nm. In total, 8 peaks were obtained and separately collected. Characterization and identification were based on HPLC-DAD, LC-MS/MS, HRMS, and NMR experiments. Spectral data for arbutin: HRMS: *m*/*z* 271.0825 (found) and *m*/*z* 271.0823 (calcd. for C_12_H_16_O_7_ /[M – H]^−^); ^1^H NMR (500 MHz, D_2_O): δ [ppm] = 3.49 (dd, ^3^*J* = 9.8/8.9 Hz, 1H–C4’), 3.53 (dd, ^3^*J* = 9.3 Hz/7.7 Hz, 1H–C2’), 3.56–3.61 (m, 1H–C3′ and 1H–C5′), 3.76 (dd, ^3^*J* = 12.5 Hz/5.6 Hz, 1H_a_–C6’), 3.93 (dd, ^3^*J* = 12.5 Hz/2.3 Hz, 1H_b_–C6’), 3.99 (d, ^3^*J* = 7.7 Hz, 1H–C1’), 6.88 (d, ^3^*J* = 9.0 Hz, 1H–C2), and 7.07 (d, ^3^*J* = 9.0 Hz, 1H–C3); and ^13^C NMR (125 MHz, D_2_O): δ [ppm] = 61.5 (C6’), 70.5 (C4’), 74.0 (C2’), 76.6 (C3′), 77.0 (C5′), 102.5 (C1’), 117.2 (C2_a/b_), 119.4 (C3_a/b_), 151.4 (C1), and 152.2 (C4).

### Enrichment of Quercetin Derivatives

About 350 mg of above middle polar prefractionated brown powder (water/methanol, 50/50) was applied to preparative flash chromatography consisting of a pump (Ismatec Reglo, Wertheim, Germany) and a glass column filled with RP-18 material (26 × 330 mm, LiChroprep RP-18 (40 – 63 μm)) connected to a fraction collector (Model 2110, Bio-Rad, Hercules, USA). A mixture of methanol/water (50/50, *v*/*v*) with the addition of formic acid (0.8 mL L^–1^) was used as the solvent with isocratic elution at 0.8 mL min^–1^. In total, 120 fractions (4 min each) were collected and examined by TLC (RP-18, water/methanol 50/50, *v*/*v*, detection with natural product reagent A at 366 nm). Fractions with the same retention factor (*R*_f_) were combined. Fractions that resulted in mixtures of various spots due to insufficient separation were refractionated on other RP-18 systems by modifying the mobile phase to water/methanol (70/30, *v*/*v*), including 0.8 mL L^–1^ of formic acid. Again, 350 mg of sample were loaded using the same running conditions, and 180 fractions were collected in total. After five runs, about 235 mg of yellowish powder were obtained, mainly containing the more polar quercetin derivatives.

### Purification of Polar Quercetin Derivatives

Above enriched polar quercetin fraction (water/methanol, 70/30) was further purified using size exclusion chromatography. Sephadex G-10 (Cytiva, Marlborough, USA) was filled into a glass column (10 mm × 350 mm), and water (100%) was used as the mobile phase. About 40 mg were applied per run using a constant flow rate of 0.7 mL min^–1^. Each minute, one fraction was collected that were monitored via TLC (RP-18, water/methanol 50/50, *v*/*v*, detection with natural products reagent A at 366 nm). Quercetin-containing fractions were then combined to result in about 150 mg of yellow powder. Further experiments showed that at least two different quercetin species were isolated. Further separation of native structures on reversed-phase systems was not possible due to their very similar retention characteristics. Thus, a polarity change was attained by permethylation of the fraction to allow separation on normal phase.

### Permethylation of Polar Quercetin Fraction

Permethylation of selected fractions containing polar quercetin derivatives was performed according to Ciucanu and Kerek.^20^ About 5.0 mg of dry material was dissolved in 0.4 mL of water-free DMSO. Then about 25 mg of finely powdered NaOH was added, and the solution was stirred for 10 min in a closed vial (4 mL). Subsequently, 100 μL of methyl iodide was injected by syringe via a septum and the reaction mixture was stirred for further 6 min. The reaction was stopped by adding 1 mL of water. The aqueous solution was then extracted three times with 2 mL of chloroform. The combined organic layers were washed three times with 10 mL of water and subsequently dried over Na_2_SO_4_. Solvents were evaporated, and the resulting dark brown and oily residue (about 120 mg) was used for chromatographic separation on normal phase.

### Separation of Permethylated Quercetin Derivatives

Permethylated quercetin derivatives were separated on a preparative silica gel column (20 × 320 mm, silica gel 60, 0.063–0.200 mm, Merck, Darmstadt, Germany) using ethyl acetate/acetone/acetic acid (3/1/0.1, *v*/*v*/*v*) as the mobile phase. The flow rate was 1.6 mL/min, and every minute one fraction was collected. About 40 mg of the permethylated fraction was applied per run to get sufficient separation of the target analytes. Fractions were controlled by TLC (silica gel 60 F_254_, same solvent, 254 nm), and fractions with material at the same *R*_f_ were combined before removal of solvents. Two pure permethylated quercetin derivatives were isolated with *R*_f_ value of 0.59 yielding 28.9 mg (compound **4**) and *R*_f_ value of 0.33 yielding 23.8 mg (compound **5**), respectively. Final structural elucidation was achieved via LC-MS/MS, HRMS, and NMR experiments.

### Partial Methylation Acetylation Analyses (PMAA) of Permethylated Quercetin Derivatives

Permethylated quercetin derivatives were hydrolyzed with 2 M TFA at 120 °C for 90 min. After removal of TFA on a vacuum centrifuge, samples were reduced with 300 μL of sodium borodeuteride (65 mg/mL) dissolved in 2 M ammonium solution to transfer the released reducing sugars to the corresponding alditols. Acetic acid (100 μL) was added to stop the reaction, followed by the addition of 450 μL of 1-methylimidazole and 3.0 mL of acetic anhydride to result in acetylation of free hydroxyl groups. 3 mL of water were added after 30 min at room temperature. Samples were extracted with dichloromethane and washed three times with water. The organic phases were transferred into glass vials, concentrated, and injected into GC-FID (Nexis GC-2030, Shimadzu, Kyoto, Japan) and GC-MS (Finnigan Trace GC ultra and Trace DSQ MS, Thermo Fisher, Dreieich, Germany) to determine the type of sugar units and their linkage to each other, following the principle of PMAA. Separation was performed on a DB-5MS column (30 m × 0.25 mm × 0.2 μm, Agilent Technologies, Santa Clara, USA) with a constant gas flow of 1.0 mL/min (linear velocity of 29.3 cm min^–1^) with helium 5.0 as the carrier gas. The temperature gradient started at 120 °C (held for 2.0 min), increasing to 200 °C with a rate of 5.0 °C min^–1^ (held for 7.0 min), then to 220 °C with 7.0 °C min^–1^ (held for 8.0 min), and finally reaching a temperature of 260 °C with 40.0 °C min^–1^ (held for 5.0 min). The inlet temperature was adjusted to 220 °C, while the ion source (EI, 70 eV) had 260 °C and the flame detector at 280 °C.

### Synthesis of 1-*O*-Caffeoyl-, 3-*O*-Caffeoyl- and 3-*O*-Feruloylglucoside

The synthesis of pure substances was performed according to the strategy of Jaiswal et al.^21,22^ After the implementation of the allyl-protected hydroxyl groups of the cinnamic acids, they were transferred to their corresponding acid chlorides. These activated structures were then coupled to 1,2/4,5-diisopropylidene glucose, and the protection groups were removed in two final steps (Pd/C and TFA). As a result, 8.5 mg of 3-*O*-caffeoyl glucoside and 17.0 mg of 3-*O*-feruloyl glucoside were obtained and unequivocally identified via NMR spectroscopy. Data for 3-*O*-caffeoyl-(α/β)-glucoside were identical to the literature.^22^ 3-*O*-Feruloyl (α/β)-glucoside: δ_H_ (400 MHz, methanol-d_4_): 7.66 (H–C3, d, ^3^*J* = 16.0 Hz, 1H), 7.19 (H–C9, d, ^3^*J* = 1.9 Hz, 1H), 7.08 (H–C5, dd, ^3^*J* = 8.2/1.9 Hz, 1H), 6.82 (H–C6, d, ^3^*J* = 8.2 Hz, 1H), 6.43 (H–C2, d, ^3^*J* = 16.0 Hz, 1H), 5.33 (H–C3′α, dd, ^3^*J* = 9.6/9.6 Hz, 0.6H), 5.17 (H–C1’α, d, ^3^*J* = 3.7 Hz, 0.6H), 5.04 (H–C3′β, dd, ^3^*J* = 9.4/9.4 Hz, 0.4H), 4.60 (H–C1’β, d, ^3^*J* = 7.8 Hz, 0.4H), 3.90 (H–C5′β, m), 3.89 (3H–C10, s, 3H), 3.66–3.86 (2H–C6’, m, 2H), 3.58 (H–C2’α, dd, ^3^*J* = 9.6/3.7 Hz, 0.6H), 3.56 (H–C4’, m, 1H), and 3.34 (H–C2’β, dd, ^3^*J* = 9.4/7.8 Hz, 0.4H). δ_C_ (100 MHz, methanol-d_4_): 169.4/161.1 (C1 α/β), 150.5 (C7), 149.4 (C8), 146.5 (C3), 127.9 (C4), 123.8 (C5), 116.2 (C6), 115.8 (C2), 111.5 (C9), 98.2 (C1’β), 94.1 (C1’α), 78.9 (C3′β), 77.7 (C5′α), 76.9 (C3′α), 74.4 (C2’β), 72.9 (C5′β), 72.1 (C2’α), 69.8/69.6 (C4’), 62.6/62.4 (C6’), and 56.5 (C10). HRMS: 357.1289 *m*/*z* found and 357.1289 *m*/*z* calcd. for C_16_H_21_O_9_ (positive mode). 1-*O*-Caffeoylglucoside was synthesized by coupling diallyl-caffeic acid chloride with 2,3,4,6-tetra-*O*-benzylglucopyranose. After purification on silica gel (petroleum ether/ethyl acetate, 1/1, *v*/*v*) both protecting groups were removed simultaneously using Pd/C and hydrogen infusion via a syringe as described above. Washing the reaction mixture with dichloromethane resulted in pure 1-*O*-caffeoyl-β-glucoside verified by HRMS and NMR: δ_H_ (400 MHz, D_2_O): 7.73 ppm (H–C3, d, ^3^*J* = 15.9 Hz, 1H), 7.18 (H–C9, s, 1H), 7.13 (H–C5, d, ^3^*J* = 8.2 Hz, 1H), 6.93 (H–C6, d, ^3^*J* = 8.2 Hz, 1H), 6.40 (H–C2, d, ^3^*J* = 15.9 Hz, 1H), 5.66 (H–C1’β, d, ^3^*J* = 7.7 Hz, 1H), 3.92 (H–C6’_A_, pseudo d, ^3^*J* = 12.4 Hz, 1H), 3.92 (H–C6’_B_, m, 1H), 3.54–3.66 (H–C2’, H–C3′, H–C5′, m, 3H), and 3.50 (H–C4’, t, ^3^*J* = 8.8 Hz, 1H); δ_C_ (100 MHz, D_2_O): 168.9 ppm (C1), 148.8 (C3), 148.7 (C8), 145.5 (C7), 127.6 (C4), 124.0 (C5), 117.2 (C6), 116.2 (C9), 114.1 (C2), 95.7 (C1’β), 77.7 (C5′β), 76.6 (C3′β), 73.0 (C2’β), 70.1 (C4’β), and 61.3 (C6’β); and HRMS: 343.1023 *m*/*z* found and 343.1024 *m*/*z* calcd. for C_15_H_19_O_9_ (positive mode).

### High-Performance Liquid Chromatography with Diode Array Detection (Quercetin Derivatives)

The HPLC system (Jasco, Pfungstadt, Germany) consisted of a pump (PU-2080 Plus) with a degasser (DG-2080–54) and a quaternary gradient mixer (LG-2080–04), a column oven (Jasco Jetstream II), an autosampler (AS-2055 Plus), and a diode array detector (MD-2015 Plus). Chromatographic separations were performed on a stainless-steel column packed with RP-18 material (Vydac 218 TP C18, 250 × 4.6 mm, 5 μm, USA) by using a flow rate of 1.0 mL min^–1^. The mobile phases used were water (solvent A) and methanol with water (7/3, *v*/*v*, solvent B). Formic acid was added to both solvents (A and B) at a concentration of 0.8 mL L^–1^. Analysis was performed at a column temperature of 25 °C using gradient elution: 10% B (10 min), increasing to 90% B (in 75 min), then to 100% B (in 5 min), held for 10 min. Detection was performed in a wavelength range between 200 and 600 nm.

### High-Performance Liquid Chromatography with Mass Spectrometry Detection (Quinic Acids, Glucaric Acids, and Glucosides)

Above HPLC apparatus was connected to an API 4000 QTrap LC-MS/MS system (Applied Biosystems/MDS Sciex, Framingham, USA) equipped with a turbo ion spray source using electrospray ionization in positive mode: sprayer capillary voltage of 4.2 kV, nebulizing gas flow of 55 mL min^–1^, heating gas of 65 mL min^–1^ at 550 °C, and curtain gas of 40 mL min^–1^. Chromatographic separations were performed on a stainless-steel column packed with RP-18 material (Vydac 218 TP C18, 250 mm × 4.6 mm, 5 μm, USA) using a flow rate of 1.0 mL min^–1^. The mobile phases used were water (solvent A) and methanol with water (7/3, *v*/*v*, solvent B). To both solvents (A and B), 0.8 mL of L^–1^ formic acid and 0.1 mmol of L^–1^ ammonium formate were added. Analysis was performed at a column temperature of 25 °C using gradient elution: 10% B (10 min), increasing to 31% B (in 20 min), then to 100% B (in 5 min), held for 7 min. For mass spectrometric detection, the multiple-reaction monitoring (MRM) mode was used in the case of quantitation, utilizing collision-induced dissociation (CID) of the protonated molecules [M + H]^+^ or the ammonium adducts [M + NH_4_]^+^ with compound-specific orifice potentials and fragment-specific collision energies (Table S1). Quantitation was based on external calibration using authentic reference material. Data for cinnamic acid derivatives obtained by LC-MS/MS showed coefficients of variation below 8.2% (*n* = 6).

### High Resolution Mass Spectrometry (HRMS)

A TripleTOF 6600–1 mass spectrometer (Sciex) was used for high-resolution mass spectrometry, which was equipped with an ESI-DuoSpray-Ion-Source (negative ion mode) and was controlled by Analyst 1.7.1 TF software (Sciex). The ESI source operation parameters were as follows: ion spray voltage: 4500 V; nebulizing gas: 60 psi; source temperature: 450 °C; drying gas: 70 psi; and curtain gas: 35 psi. Data acquisition was performed in the MS1 TOF mode and scanned from 100 to 1500 Da with an accumulation time of 50 ms.

### Nuclear Magnet Resonance Spectroscopy (NMR)

NMR spectra were recorded on a Varian Unity Inova 500 instrument operating at 500 or 400 MHz for ^1^H and at 125 or 100 MHz for ^13^C, respectively. SiMe_4_ was used as a reference for calibrating the chemical shift.

### Statistical Evaluation

Analyses of all given concentrations were performed at least in a 6-fold determination for each individual sample and parameter. Confidence intervals were calculated with a probability of 95%. Quantitation was achieved through external calibration with regression coefficients *R*^2^ between 0.9973 and 0.9992, proving an adequate linear fit for quantitation. Statistical evaluation was performed by the use of SigmaPlot software (Version 14.0 Build 14.0.3.192, Systat Software Inc.). Validation data for quantitation methods is given in Table S2.

## Results and Discussion

### HLPC-DAD Screening of the SG Crude Extract

**Figure 2:**
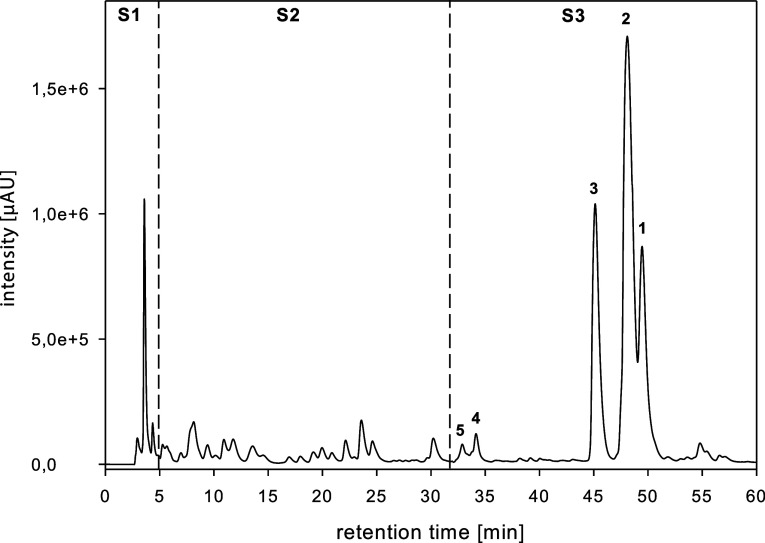
HPLC-UV chromatogram of a crude extract of SG leaves using acetone/water (7/3), chromatogram measured at 280 nm (S1), 320 nm (S2), and 350 nm (S3).

Extraction of dried leaves of *Solanum glaucophyllum* Desf. (SG) with a mixture of acetone and water (7/3) resulted, after evaporation and freeze-drying, in a brown powder with an overall yield of 26%. The SG crude extract was dissolved in methanol and water (1:1) and analyzed via HPLC-DAD for the first screening of phenolic compounds. A characteristic chromatogram is shown in Figure 2. Evaluation of the absorption maxima of all detected peaks led to the division into three sections. SectionS1 was characterized by substances with absorption maxima of about 280 nm, indicating simple aromatic systems, such as those known for arbutin (*p*-hydroquinone glucoside), which have already been described for SG. This section was limited to a small retention time region from 2.5 to 5.0 min, indicating the presence of very polar substances. A second section S2 was observed between 5.0 and 32.0 min, where all peaks had absorption maxima around 310 to 320 nm. Thus, especially cinnamic acid derivatives were expected in this region. Section S3 with more lipophilic compounds was observed between 32.0 and 60.0 min. Here, all major peaks (1–5) had absorption maxima at about 350 nm, suggesting the presence of flavon-3-ol derivatives. Total acidic hydrolyses of the crude extract allowed the identification of predominantly quercetin, determined at *t*_R_ = 61.68 min, with an amount of 91.5%. Kaempferol (*t*_R_ = 67.88 min) and isorhamnetin (*t*_R_ = 72.40 min) reached only 4.4% and 4.1%, respectively. These low concentrations were contrary to literature, where isorhamnetin and kaempferol derivatives were also isolated from SG.^10^ This might be due to the special variety, Hervit 153, of SG used in the present investigation. Before hydrolysis, no flavon-3-ol aglycones were detected.

Starting from the crude extract, various chromatographic techniques were used for enrichment, separation, and purification to allow comprehensive identification and quantitation of phenolic compounds. Figure 1 shows the strategy that allowed the isolation of 8 pure substances from the SG crude extract, 5 of them were quercetin glycosides (**1**–**5**) and the remaining were identified as arbutin and two cinnamoyl glucaric acid derivatives (**GA1** and **GA2**).

### Isolation, Structural Elucidation, and Quantitation of Quercetin Derivatives

**Figure 3:**
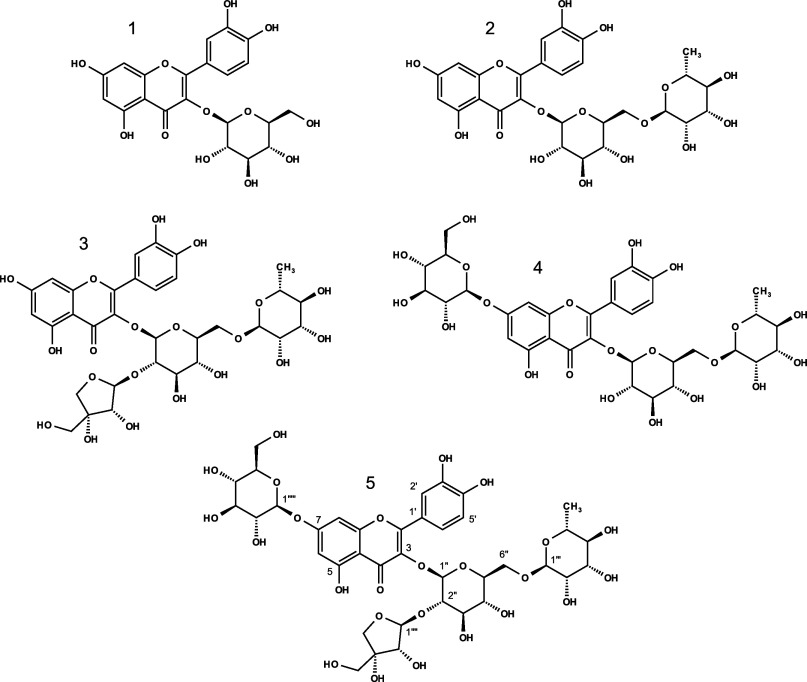
Chemical structures of five isolated quercetin (Q) derivatives from SG extract: isoquercetin **1**, rutin **2**, apiosyl-rutin **3**, 7-*O*-β-glucosyl-rutin **4**, and 7-*O*-β-glucosyl-α-apiosyl-rutin **5**.

The combination of two different reversed-phase chromatographies led to the isolation of 3 pure substances (Figure 2 and 3, compounds **1**–**3**). Mass spectrometric experiments revealed the same backbone, underlining quercetin as the aglycone due to the characteristic fragment ion of 303 *m*/*z* for all three compounds.^23^ The addition of one glucose unit was found with a quasi-molecular ion of 465.3 *m*/*z* [M + H]^+^ for compound **1** leading to the verification of isoquercetin.^24^ Peak **2**, with a pseudo-molecular ion of 611.3 *m*/*z* [M + H]^+^ was identified as rutin by addition of rutinose.^23^ Peak **3** showed a [M + H]^+^ of 743.3 *m*/*z* confirming the addition of a pentose, such as apiose, to rutin. Follow-up HRMS, as well as 1D- and 2D-NMR experiments, unequivocally substantiated the identification of isoquercetin (quercetin-3-*O*-glucoside), rutin (quercetin-3-*O*-rutinoside), and apiosyl-rutin (quercetin-2’’-*O*-apiosyl-3-*O*-rutinoside).^23^ These metabolites were already identified in SG; thus, our experiments confirmed the published compounds with detailed spectroscopic data summarized in Table 1.^10^

**Table 1:** UV Spectral Data and Mass Spectrometric Data of Quercetin Derivatives^a^

no.	compound	HPLC-DAD λ_max_ (nm)	HRMS/ESI(−) [M – H]^−^ (*m*/*z*) found	calculated	HPLC/ESI(+)-MS^2^ (*m*/*z*)	amount in leaves of SG (% d.m.)
1	Isoquercetin	350, 252	463.0865	463.0875	465.3, 303.3	1.12 ± 0.04
2	Rutin	350, 253	609.1449	609.1461	611.3, 465.0, 302.9	1.74 ± 0.04
3	Apiosyl-rutin	350, 253	741.1883	741.1884	743.3, 611.3, 465.2,	1.00 ± 0.02
					303.3	
4	7-*O*-Glucosyl-rutin	348, 253	771.1985	771.1989	773.3, 627.3, 465.5,	0.17 ± 0.01
					303.5	
5	7-*O*-Glucosyl-apiosyl-rutin	348, 255	903.2401	903.2412	905.3, 773.4, 627.7, 465.5, 303.4	0.13 ± 0.01

a d.m.: dry matter

During the second fractionation step on the reversed phase (methanol/water, 50/50), an enrichment of two other quercetin derivatives was achieved (Figures 2 and 3, compounds **4** + **5**). This suggestion was first based on the typical UV–vis spectra (λ_max_ at 350 nm), reaction with natural products reagent A to produce orange/yellow spots after TLC and the characteristic fragment ion of 303 *m*/*z* for quercetin during LC-MS/MS experiments. Both compounds were much more polar compared to the above isolated structures, as evidenced by their HPLC retention times, as shown in Figure 2, and their TLC-*R*_f_ value of 0.56, compared to, e.g., apiosyl-rutin with an *R*_f_ value of 0.30 (TLC, RP-18, methanol/water, 50/50, *v*/*v*). However, the polar quercetin derivatives **4** and **5** coeluted with each other and other plant metabolites, such as quinic acids, and had to be further purified. As trials with a third RP-18 chromatography failed and still showed unsatisfactory impurities in the form of chlorogenic acids, the mixture was purified on Sephadex G10 material by size exclusion chromatography. As known from above LC-MS/MS experiments, the targeted polar quercetin derivatives had molecular weights above 750 g/mol, while chlorogenic acids, with a molecular weight of 354 g/mol, are comparatively much smaller molecules. Indeed, while this step removed other impurities, the two quercetin derivatives could not be separated, apparently due to their very similar polarity and chromatographic behavior. Nevertheless, HRMS experiments (negative mode) of the resulting mixture verified our suggestion that glucosyl-rutin **4** and glucosyl-apiosyl-rutin **5** are present in the SG (Table 1). Molecular formulas were calculated from experimental masses of 771.1983 *m*/*z* leading to C_33_H_39_O_21_ ([M – H]^−^) and 903.2401 *m*/*z* leading to C_38_H_47_O_25_ ([M – H]^−^), while experimental and theoretical masses differed by 0.8 and 1.2 ppm, respectively. Compared to rutin and apiosyl-rutin, the elemental composition increased by C_6_H_10_O_5_, which verified an additional hexose unit (C_6_H_10_O_5_ + H_2_O = C_6_H_12_O_6_). Substances with the very same molecular formula were already reported in the literature. For example, Wei et al. determined a quercetin tetra-glycoside in *Aesculus chinensis*.^25^ In that report, quercetin-3′-*O*-glucosyl-(2’’-*O*-xylosyl-3-*O*-rutinoside) was isolated from plant seeds (Aescuflavoside). 2D-Experiments unequivocally proved that, in comparison to apiosyl-rutin, one more glucose unit was linked at C3′ of the B-ring of quercetin.^25^ Gómez-Romero et al. identified a rutin hexoside (quercetin-*O*-hexosyl-3-*O*-rutinoside) during HRMS profiling of tomato fruits with the same chemical formula of C_33_H_40_O_21_ as we found in SG.^16^ Unfortunately, there was no specific structural evaluation due to the missing isolation efforts. Others defined the same molecular formula as delphinidin-3-*O*-rutinoside-5-*O*-glucoside or just as quercetin-3-*O*-trisaccharide.^26,27^ This underlines the limitation of stand-alone HRMS experiments that easily allow the suggestion of numerous hypothetical substances based on chemical formulas. However, for absolute structural elucidation, it is mandatory to isolate compounds for full spectroscopic and spectrometric characterization.

**Figure 4:**
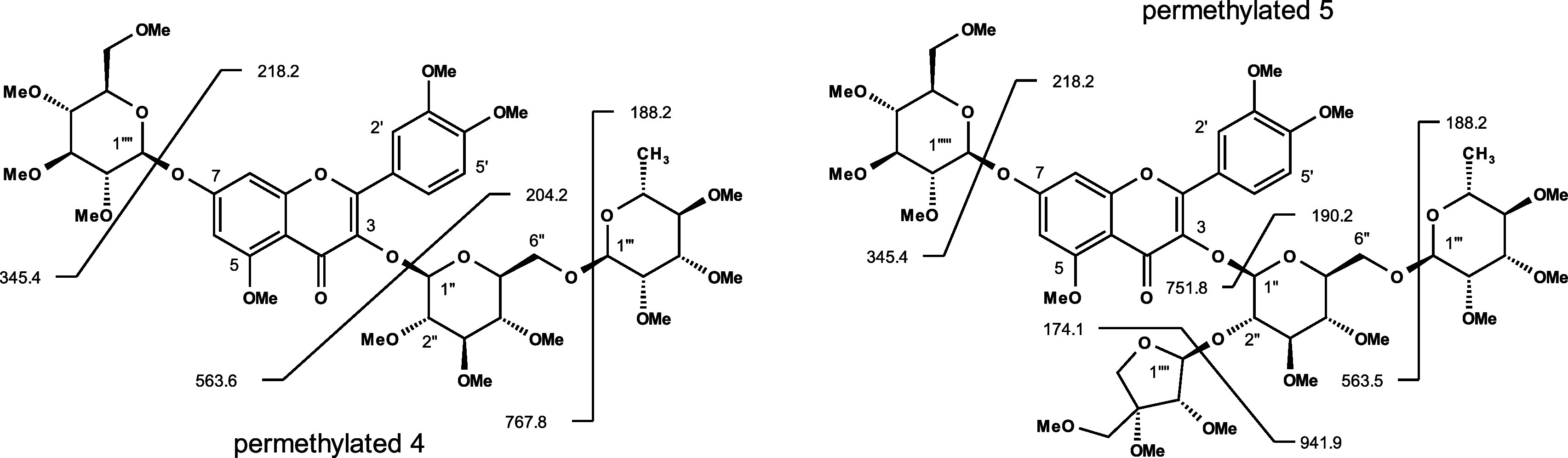
LC-MS/MS fragmentation of permethylated 7-*O*-β-glucosyl-rutin **4** and 7-*O*-β-glucosyl-α-apiosyl-rutin **5**.

Consequently, in the present work, the enriched quercetin fraction was permethylated according to Ciucanu and Kerek.^20^ Methylation led to a significant polarity shift of the native, very polar substances; e.g., LC-MS/MS retention times changed from 8.58 to 23.42 min for compound **4** and from 7.37 to 21.47 min for compound **5**. The quasi-molecular ion of compound **4** changed from 773.3 to 955.6 *m*/*z* [M + H]^+^ verifying the implementation of 13 methoxy groups and proving the completeness of permethylation (13x 14.026 *m*/*z* = 182.34 *m*/*z*). The same was true for the tetra-glycoside **5**. Here, the permethylated quasi-molecular ion [M + H]^+^ had a mass of 1115.8 *m*/*z* showing an increase of 210.5 *m*/*z* compared to the native structure with 905.3 *m*/*z* (15x 14.026 *m*/*z* = 210.39 *m*/*z*). MS/MS experiments of both substances proved that two positions of the quercetin backbone were coupled to the sugar units. In comparison to the fragmentation of the native structure with the typical quercetin fragment ion at *m*/*z* 303, permethylation now led to 345 *m*/*z* (Figure 4). This corresponds to an increase of 42 *m*/*z* representing the conversion of three hydroxy groups to the corresponding methoxy groups. As native quercetin has 5 hydroxyl groups located in the molecule, two of them are glycosylated and consequently not derivatized during permethylation. LC-MS/MS experiments of the permethylated quercetin derivatives allowed further structural insights to the sugar connectivity. First, fragmentation of the permethylated triglycoside compound **4** with [M + H]^+^ of 956.0 *m*/*z* resulted in 767.8 *m*/*z* caused by the loss of a rhamnose unit modified with 3 methoxy groups (Rha-OMe_3_, −188.2). Subsequent loss of a three-times modified glucose (Glc-OMe_3_, −204.2) unit led to the second observed fragment of 563.6 *m*/*z*. This fragmentation pattern revealed that this glucose unit has to be linked to quercetin and in parallel to rhamnose, as known for quercetin-rutinoside (rutin). For compound **4**, the last observed fragment of 345.4 *m*/*z* resulted from a loss of 218.2 *m*/*z* that solely can be explained by the elimination of a 4 times methylated glucose unit. Consequently, the second glucose unit has to be located on another binding site of the quercetin backbone, supporting the above presence of a 3-fold methylated quercetin fragment ion (345 *m*/*z*). Comparable fragmentation was observed for the permethylated tetra-glycoside compound **5** (1116.0 *m*/*z*). First, a fragment ion of 941.9 *m*/*z* was produced after the loss of a three-times methylated apiose (Api-OMe_3_, −174.1 *m*/*z*). Ions with 751.8 and 563.5 *m*/*z* were generated after cleavage of two-times methylated glucose (Glc-OMe_2_, −190.2) and three-times methylated rhamnose (Rha-OMe_3_, −188.2), respectively. This fragmentation pattern was a clear indication for a connectivity as observed for quercetin-apiosyl-rutinoside, where glucose represents the binding site for apiose (1’’’’→2’’), rhamnose (1’’’→6’’), and quercetin (3-*O*). The last fragment was explained again by the loss of a terminal glucose (Glc-OMe_4_, −218.2) from 563.5 to 345.4 verifying the isolation of a quercetin-glucosyl-apiosyl-rutinoside derivative. Methylated structures were supported by HRMS and, in the case of compound **4** exemplary via partial methylation acetylation analyses (PMMA), verifying the connectivity of all present sugar units (Table 2 and Figure S8).

**Table 2:** ^1^H and ^13^C NMR Spectroscopic Data of Permethylated 7-*O*-β-Glucosyl-Rutin (4) and Permethylated 7-*O*-β-Glucosyl-α-Apiosyl-Rutin (5)

	methylated 7-*O*-β-glucosyl-rutin **4**		methylated 7-*O*-β-glucosyl-α-apiosyl-rutin **5**	
HRMS (found)	955.4175		1115.4894	
HRMS (calcd.)	955.4169 ([M + H] = C_46_H_67_O_21_)		1115.4905 ([M + H] = C_53_H_79_O_25_)	
C/H	δ ^1^H [ppm]	δ ^13^C [ppm]	δ ^1^H [ppm]	δ ^13^C [ppm]
Quercetin				
**2**	---	154.3	---	154.5
**3**	---	136.2	---	136.1
**4**	---	173.5	---	
**5**	---	161.2	---	161.5
**6**	6.49 (d, 1H, ^4^*J* = 2.2 Hz)	96.9	6.48 (d, 1H, ^4^*J* = 2.2 Hz)	96.1
**7**	---	161.3	---	161.2
**8**	6.64 (d, 1H, ^4^*J* = 2.2 Hz)	96.3	6.64 (d, 1H, ^4^*J* = 2.2 Hz)	96.1
**9**	---	158.4	---	158.5
**10**	---	110.4	---	110.4
**1’**	---	123.4	---	123.4
**2’**	7.80 (d, 1H, ^4^*J* = 2.1 Hz)	112.5	8.12 (d, 1H, ^4^*J* = 2.1 Hz)	113.0
**3′**	---	151.0	---	151.0
**4’**	---	148.4	---	148.4
**5′**	6.94 (d, 1H, ^3^*J* = 8.6 Hz)	110.7	6.95 (d, 1H, ^3^*J* = 8.6 Hz)	110.3
**6’**	7.68 (dd, 1H, ^3^*J* = 8.6/^4^*J* = 2.1 Hz)	122.2	7.57 (dd, 1H, ^3^*J* = 8.6/^4^*J* = 2.1 Hz)	121.1
C3′-O**CH**_**3**_	3.95 (s, 3H)	55.8	3.94 (s, 3H)	56.0
C4’-O**CH**_**3**_	3.95 (s, 3H)	56.1	3.99 (s, 3H)	56.2
C5–O**CH**_**3**_	3.96 (s, 3H)	56.4	3.95 (s, 3H)	56.2
**3-*****O*****-β-Glucose**				
**1’’**	5.71 (d, 1H, ^3^*J* = 7.5 Hz)	100.6	5.78 (d, 1H, ^3^*J* = 7.6 Hz)	101.1
**2’’**	3.19 (m)	84.5	3.54 (m)	75.3
**3′’**	3.27 (m)	86.5	3.29 (m)	86.5
**4’’**	2.95 (m)	80.1	3.52 (m)	81.1
**5′’**	3.36 (m)	74.5	3.23 (m)	79.1
**6’’A**	3.72(m)	66.9	3.67(m)	66.3
**6’’B**	3.36 (m)		3.44(m)	
C2’’-O**CH**_**3**_	3.61 (s, 3H)	60.0	---	---
C3′’-O**CH**_**3**_	3.66 (s, 3H)	60.7	3.67 (s, 3H)	61.0
C4’’-O**CH**_**3**_	3.49 (s, 3H)	60.3	3.61 (s, 3H)	60.1
**6’’-***O***-α-Rhamnose**				
**1’’’**	4.64 (d, 1H, ^3^*J* = 1.8 Hz)	97.6	4.67 (d, 1H, ^3^*J* = 1.8 Hz)	97.4
**2’’’**	3.31 (m)	77.4	3.35 (m)	77.1
**3′’’**	3.24 (m)	81.0	3.32 (m)	80.8
**4’’’**	2.92 (m)	82.1	3.02 (m)	82.0
**5′’’**	3.36 (m)	67.9	3.44 (m)	67.9
**6’’’**	1.07 (d, 3H, ^3^*J* = 6.2 Hz)	17.7	1.22 (d, 3H, ^3^*J* = 6.3 Hz)	17.7
C2’’’-O**CH**_**3**_	3.23 (s, 3H)	58.7	3.26 (s, 3H)	58.7
C3′’’-O**CH**_**3**_	3.34 (s, 3H)	57.6	3.38 (s, 3H)	57.6
C4’’’-O**CH**_**3**_	3.41 (s, 3H)	60.7	3.48 (s, 3H)	60.9
**2’’-***O***-Apiose**	---	---		
**1’’’’**	---	---	5.62 (d, 1H, ^3^*J* = 1.8 Hz)	100.1
**2’’’’**	---	---	3.85 (m)	78.7
**3′’’’**	---	---	---	75.7
**4’’’’**	---	---	3.80 (m)	60.7
			3.69 (m)	
**5′’’’**	---	---	3.48 (m)	74.6
C2’’’’-O**CH**_**3**_	---	---	3.56 (s, 3H)	60.1
C3′’’’-O**CH**_**3**_	---	---	3.52 (s, 3H)	61.1
C5′’’’-O**CH**_**3**_	---	---	3.36 (s, 3H)	57.2
**7-***O***-β-Glucose**				
**1’’’’’**	4.94 (d, 1H, ^3^*J* = 7.1 Hz)	100.9	4.95 (d, 1H, ^3^*J* = 7.1 Hz)	100.5
**2’’’’’**	3.31 (m)	83.5	3.31 (m)	83.4
**3′’’’’**	3.30 (m)	86.1	3.30 (m)	83.9
**4’’’’’**	3.22 (m)	79.2	3.22 (m)	79.2
**5′’’’’**	3.45 (m)	75.3	3.48 (m)	75.3
**6’’’’’A**	3.61 (m)	71.1	3.63(m)	75.3
**6’’’’’B**	3.56(m)		3.58(m)	
C2’’’’’-O**CH**_**3**_	3.65 (s, 3H)	60.8	3.66 (s, 3H)	60.7
C3′’’’’-O**CH**_**3**_	3.65 (s, 3H)	61.0	3.57 (s, 3H)	58.9
C4’’’’’-O**CH**_**3**_	3.55 (s, 3H)	60.5	3.56 (s, 3H)	60.5
C6’’’’’-O**CH**_**3**_	3.36 (s, 3H)	59.4	3.37 (s, 3H)	59.4

Most importantly, permethylation now allowed for clear separation of both quercetin derivatives on normal-phase chromatography. The permethylated quercetin-triglycoside **4** had a *R*_f_ value of 0.56, while the permethylated quercetin-tetra-glycoside **5** was found at *R*_f_ 0.32 on TLC, resulting in two pure substances with amounts of about 25 mg for full structural elucidation via 1D- and 2D-NMR spectroscopy. Complete assignment of the novel substances is summarized in Table 2 (Figures 4 and S1 and S2). Obviously for the present multiglycosylated compounds, ^1^H NMR leads to superimposed signals, often hampering the differentiation of multiplets. Nevertheless, with 2D-NMR experiments, an assignment of all protons and carbons was possible. For example, for methylated **4**, starting from the anomeric proton of glucose coupled 3-*O*- to quercetin (H–C1’’, 5.71 ppm), H–C2’’ was identified via H,H–COSY because this is the only adjacent proton via three bonds (H–C2’’, 3.19 ppm). HSQC then allowed the identification of the corresponding C2’’ (75.3 ppm). Next, H,H–COSY led via ^3^*J*-coupling of H–C2’’ to the identification of H–C3′’ (3.27 ppm). These first assignments were then extended by HMBC experiments that allowed the H-to-C correlation over three bonds. E.g., H–C1’’ showed correlation to 136.1 ppm (C3 of quercetin) and to 74.5 ppm, verified as C5′’ of the same glucose unit. APT experiments also supported our findings by allowing differentiation between C- or CH_2_–groups and CH- or CH_3_-groups. This step-by-step procedure led to unequivocal assignment of both novel structures representing rutin and apiosyl-rutin with an additional glucose linked to C7 of the quercetin backbone. This position was clearly set in HMBC experiments, where anomeric H1 (4.94 ppm) of glucose correlated to C7 of quercetin (161.2 ppm) for both molecules. Coupling constants (^3^*J*_H,H_) for all anomeric protons (H–C1) of coupled sugar units allowed the assignment of α- or β-configuration. Here, coupling constants of 7.1 or 7.5 Hz showed that 3-*O*- and 7-*O*-glucose were present in β-configuration, while α-rhamnose and α-apiose were determined according to a low coupling constant of 1.8 Hz. 7-*O*-β-Glucosyl-rutin **4** was found for the first time in SG but was already reported for other *Solanaceae* such as tomatoes or potatoes or also in Calafate fruits (*Berberis microphylla*).^18,23,24^ Tomczyk and Gudej found **4** in *Ficaria verna* flowers and generated detailed NMR data, thus allowing a comparison to our findings that were very similar, even if our structure was analyzed permethylated.^28^ In contrast, quercetin-7-*O*-β-glucosyl-3-*O*-β-(2’’-*O*-α-apiosyl)-rutinoside **5** is a novel substance that was not described in literature before, as checked by database research for C_38_H_48_O_25_. As this structure is so far unique to *Solanum glaucophyllum* Desf., we would like to call it “glaucophylloside”. A similar quercetin-tetra-glycoside was isolated from *Aesculus chinensis* but here the second glucose unit was coupled to 3′-*O*-position at the B-ring of the quercetin backbone.^25^ The published NMR data by Wei et al. were very similar to our findings, verifying the apiosyl-rutinoside backbone. However, the biggest difference was the coupling of the anomeric proton of the terminal glucose unit (H–C1, 4.94 ppm).^25^ While they observed an H–C correlation to 145.1 ppm (aromatic C3′, B-ring), we found a coupling to 161.2 ppm that is unequivocally associated with C7 of the A-ring.^25^ Another publication isolated quercetin-3-*O*-rutinoside-7-*O*-xylosylglucoside from *Paederia scandens* var. *mairei* that was verified via ^1^H- and ^13^C NMR.^29^ This configuration was also excluded for the present isolated substance due to our NMR data and the MS fragmentation as extensively discussed for the permethylated substances above.

After identification and isolation of the major 5 flavon-3-ol derivatives from SG, they were quantitated via HPLC-UV with external calibration based on authentic reference material (Table 1). The three dominant quercetin derivatives, **1**, **2**, and **3**, had concentrations of 1.12, 1.74%, and 1.00% based on dried leaf material. According to this, a ratio of about 0.6/1.0/0.6 was observed, which was comparable to other *Solanaceae*.^16,27^ The newly found quercetin tri- and tetra-glycosides (**4** and **5**) were found at concentrations of 0.17% and 0.13%, respectively, representing about 4% of the total quercetin derivative amount.

### Isolation, Structural Elucidation, and Quantitation of Glucaric Acid (GA) Derivatives

**Figure 5:**
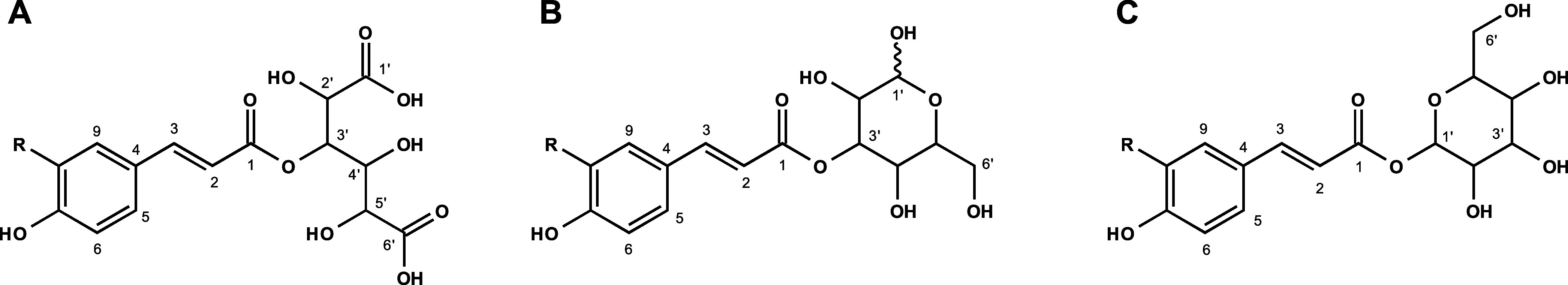
Chemical structures of isolated or synthesized cinnamic acid derivatives that were identified and quantitated in SG extracts. (A) *R*=H – 3-*O*-*p*-coumaroyl glucaric acid, *R*=OH – 3-*O*-caffeoyl glucaric acid, and *R*=OCH_3_ – 3-*O*-feruloyl glucaric acid, (B) *R*=H – 3-*O*-*p*-coumaroyl glucoside, *R*=OH – 3-*O*-caffeoyl glucoside, and *R*=OCH_3_ – 3-*O*-feruloyl glucoside, and (C) *R*=OH – 1-*O*-caffeoyl glucoside.

**Figure 6:**
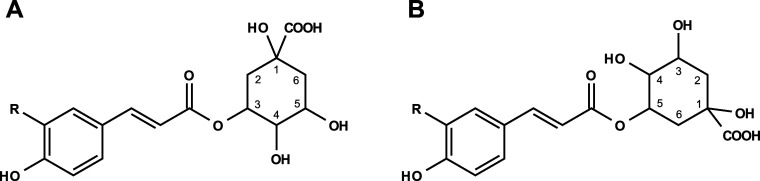
Chemical structures of identified and quantitated cinnamoyl quinic acids found in SG extract. (A) *R*=H – 3-*O*-*p*-coumaroyl quinic acid, *R*=OH – 3-*O*-caffeoyl quinic acid, and *R*=OCH_3_ – 3-*O*-feruloyl quinic acid, (B) *R*=H – 5-*O*-*p*-coumaroyl quinic acid, *R*=OH – 5-*O*-caffeoyl quinic acid, and *R*=OCH_3_ – 5-*O*-feruloyl quinic acid.

Material from the polar prefractionation step (water/methanol, 95/5) was further separated on a preparative HPLC-UV system. Eight fractions were collected based on the UV chromatogram monitored at 320 nm. Analyses via LC-MS/MS of individual fractions revealed that three of them contained pure substances (arbutin, **GA1**, and **GA2**; see Figure 1) while all others showed mixtures of at least two substances. Further efforts for separation were not successful due to very similar polarities. Arbutin was unequivocally identified via HRMS and NMR data that were completely identical to literature.^10^ LC-MS/MS analyses of the other both pure substances led to pseudo molecular peaks of 373.2 [M + H]^+^, 390.2 [M+NH_4_]^+^, and 395.2 [M + Na]^+^ for **GA1**, and 357.1 [M + H]^+^, 374.0 [M+NH_4_]^+^ and 730.4 [2M+NH_4_]^+^ for **GA2**. Fragmentation of [M+ NH_4_]^+^ in an MS/MS experiment led to 163.3, 145.4, 135.5, and 117.3 *m*/*z* for **GA1**. This indicated the presence of caffeic acid because of the very typical fragmentation pattern known from literature.^30^ [M+ NH_4_]^+^ of **GA2** resulted in fragment ions of 147.2 and 119.1 *m*/*z* verifying *p*-coumaric acid as the phenolic component.^31^ In both cases, the counterpart was determined with a nominal mass of 210 *m*/*z*. A review of the literature verified that glucaric acid was the binding partner for the cinnamic acids.^32^ However, reports on mass spectrometric screening of tomatoes or calafate berries revealed the presence of up to 5 different caffeoyl glucaric acid isomers with the observed molecular weight of 372.1 g/mol.^23,33^ HRMS experiments confirmed the elemental compositions of C_15_H_15_O_11_ for **GA1** and C_15_H_15_O_10_ for **GA2**. High-resolution MS^3^ data for the α-fragment ion of glucaric acid (209.0305) gave *m*/*z* values of 191.0200 (C_6_H_7_O_7_, loss of water), 147.0303 (C_5_H_7_O_5_, additional loss of carbon dioxide), 133.0147, and 85.0299, respectively, completely compliant with fragmentation data for glucaric acid from the literature.^34,35^ Final structure elucidation via NMR spectroscopy is given in Table 3 (Figures 5 and S3 and S4). Proton in position 3′ or 4’ of glucaric acid (H–C3′/4’ at 5.13 ppm) showed H–C HMBC correlation to 168.9 and 174.9 ppm over three bonds (**GA1**). These signals represent the carbonyl carbons of one terminal carboxylic acid of glucaric acid (C1’) and the carbonyl group of the attached caffeic acid (C1). H,H–COSY experiments revealed that the proton H–C3′ has at least 2 other protons adjacent. Thus, 2-*O*- or 5-*O*-caffeoyl glucaric acid was unequivocally excluded. Unfortunately, the 3- or 4-*O*-isomer as well as the 2- or 5-*O*-isomer cannot practically be differentiated due to their pseudo-symmetry.^32^ Ruiz et al. also isolated 2 caffeoyl glucaric acid isomers from tomatoes and evaluated them as 3-*O*- and 4-*O*-isomers via ^1^H NMR but failed to specify which peak corresponded to which compound.^33^ Contrary, Strack et al. reported on the enzymatic synthesis of caffeoyl glucaric acid starting from 5-*O*-caffeoyl quinic acid. Analyses of the resulting product mainly revealed the formation of 2-*O*- or 5-*O*-caffeoyl glucaric acid. NMR experiments did not allow distinction between position 2 or 5 but a clear differentiation to the 3- or 4-*O*-isomer was discussed due to the multiplicity of proton signals and low field shift of H–C2’ or H–C5′.^32^ However, in parallel to the present data caffeic acid of the isolated substance showed a characteristic coupling constant of 16.0 Hz (^3^*J*) for both protons at the nonaromatic double bond that proved *trans* configuration (Figure 6).

**Table 3:** ^1^H and ^13^C NMR Spectroscopic Data of Isolated Coumaroyl and Caffeoyl Glucaric Acid

	3- or 4-*O*-*p*-*trans*-coumaroyl glucaric acid GA2			3- or 4-*O*-*trans*-caffeoyl glucaric acid GA1		
HRMS (found/calcd.)	355.0627/355.0630 ([M – H] = C_15_H_15_O_10_)			371.0622/371.0620 ([M – H] = C_15_H_15_O_11_)		
C/H	δ ^1^H [ppm]	δ ^13^C [ppm]	connectivity	δ ^1^H [ppm]	δ ^13^C [ppm]	connectivity
Cinnamic acid						
**1**	---	168.8		---	168.9	
**2**	6.48 (d, 1H, ^3^*J* = 16.0 Hz)	114.6		6.36 (d, 1H, ^3^*J* = 16.0 Hz)	114.2	**C**4^a^
**3**	7.77 (d, 1H, ^3^*J* = 16.0 Hz)	147.1	C1^a^	7.60 (d, 1H, ^3^*J* = 16.0 Hz)	146.3	**C**1^a^
**4**	---	127.6		---	127.0	
**5**	7.51 (d, 2H, ^3^*J* = 8.6 Hz)	131.0		7.03 (d, 1H, ^3^*J* = 8.2 Hz)	122.8	**C7**^a^, **H**-C6^b^
**6**	6.84 (d, 2H, ^3^*J* = 8.6 Hz)	116.6		6.81 (d, 1H, ^3^*J* = 8.2 Hz)	116.2	**H**-C5^a^
**7**	---	161.3		---	147.2	
**8**	äquivalent to 6	---		---	146.7	
**9**	äquivalent to 5	---		7.10 (s, 1H)	115.0	**C**7^a^
3/4-*O*-Glucaric Acid						
**1’ a**	---	183.2		---	174.9	
**2’ b**	4.46 (br d, 1H, ^3^*J* = 8.7 Hz)	75.0		4.31 (br s, 1H)	70.5	
**3′ c**	5.43 (s, 1H)	74.0	H–C4’^b^	5.13 (br s, 1H)	74.4	**C**1^a^, **C**1’^a^, **H**-C4’^b^
**4’ c**	4.29 (br d, 1H, ^3^*J* = 7.6 Hz)	71.7	H–C3’^b^, H–C5’^b^	4.11 (d, 1H, ^3^*J* = 10.0 Hz)	70.5	**H**-C3’“^b^,’**H**-C5”^b^
**5′ b**	4.02 (br d, 1H, ^3^*J* = 7.6 Hz)	72.6	H–C4’^b^	3.90 (d, 1H, ^3^*J* = 10.0 Hz)	71.2	**H**-C4’^b^
**6’ a**	---	183.2		---	174.9	

a via HMBC (^3^J H-to-C-connectivity).

b via H,H–COSY (^2^J H-to-H-connectivity), a/b/c signals with same letter can be interchanged.

**Figure 7:**
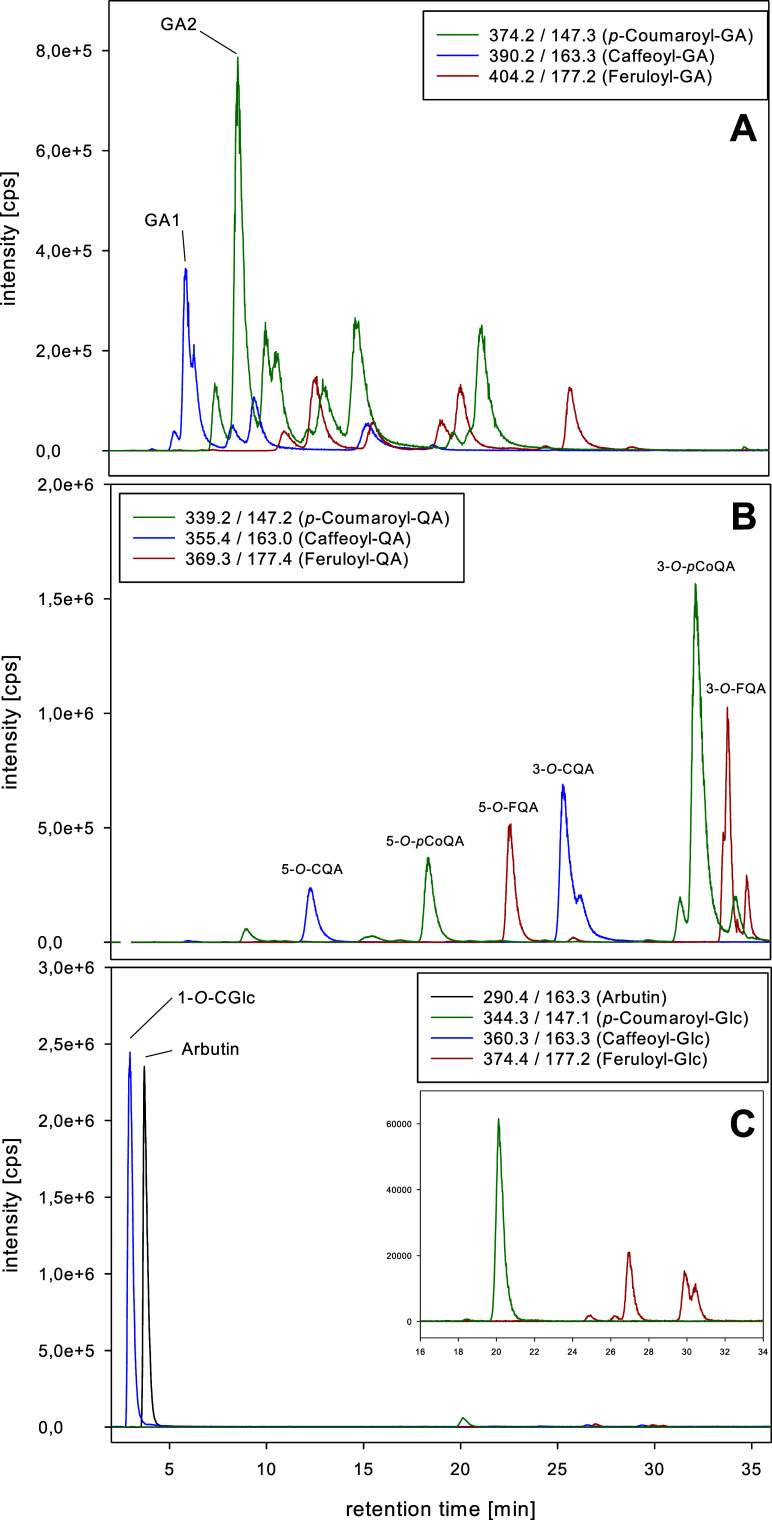
MRM chromatograms obtained for hydroquinone and cinnamic acid derivatives in SG crude extract: (A) MRM for *p*-coumaroyl-, caffeoyl-, and feruloyl glucaric acids, (B) MRM for *p*-coumaroyl-, caffeoyl-, and feruloyl quinic acids, and (C) MRM for arbutin, *p*-coumaroyl-, caffeoyl-, and feruloyl glucosides.

This is also valid for the isolated *trans*-*p*-coumaroyl glucaric acid derivative (**GA2**). The obtained NMR data can be compared to literature where 2-*O*- or 5-*O*-*p*-coumaroyl glucaric acid was isolated from orange peel (*Citrus*).^36^ However, as for the above caffeoyl derivative, the isolated compound is 3- or 4-*O*-*p*-coumaroyl glucaric acid especially because of H,H–COSY correlation experiments, as given in Table 3. Again, differentiation between the 3-*O*- and 4-*O*-isomer was not possible. In future, this isomeric issue should be clarified by regiospecific syntheses. With the isolated authentic reference material, a sensitive LC-MS method was developed (Table S1). Analyses of the crude extract of SG verified more than 4 signals based on the optimized MRM method for the [M+NH_4_]^+^ 374.2 and 390.2, respectively (Figure 7A). This must be owed to the published occurrence of 2-*O*- and 5-*O*-isomers, but most likely also to stereoisomers.^23,32,36^ In addition, an MRM transition method for feruloyl glucaric acids was simulated. Thus, the calibration curve from the isolated 3/4-*O*-isomers leads to correct quantitation for these substances, while all other data must be evaluated as semiquantitative, although similar mass-spectrometric characteristics can be expected (Table 4).

**Table 4:** Quantitation of All Determined Cinnamic Acid Derivatives in SG (*p*Co – *p*-Coumaroyl, C – Caffeoyl, F – Feruloyl)

compound	retention time [min]	MRM quantifier/qualifier (pos. mode)	amount in leaves of SG (ppm per d.w.)
Glucaric acids (GA)			
3/4-*O*-*p*CoGA	8.71	374.2 → 147.3/119.3	2450 ± 70
5 Other isomers sum of all *p*CoGA	7.59, 10.28, 13.31, 14.98, 21.36	374.2 → 147.3/119.3	400 – 1200
			6400 ± 200
3/4-*O*-CGA	6.04	390.2 → 163.3/145.4	1250 ± 50
3 Other isomers sum of all CGA	6.46, 9.63, 15.48	390.2 → 163.3/145.4	150 – 360
			2010 ± 90
X-*O*-FGA	12.82	404.2 → 177.2/145.2	625 ± 25
5 Other isomers sum of all FGA	11.25, 15.76, 19.37, 20.31, 25.97	404.2 → 177.2/145.2	150 – 500
			2200 ± 80
Quinic acids (QA)			
5-*O*-*p*CoQA	17.97	339.2 → 147.2/119.5	72 ± 4
3-*O*-*p*CoQA	32.01	339.2 → 147.2/119.5	355 ± 20
5-*O*-CQA	12.47	355.4 → 163.0/145.2	157 ± 3
3-*O*-CQA	25.50	355.4 → 163.0/145.2	560 ± 15
5-*O*-FQA	23.32	369.2 → 177.4/145.2	340 ± 10
3-*O*-FQA	35.58	369.2 → 177.4/145.2	580 ± 35
Glucosides (Glc)			
*p*CoGlc	20.13	344.3 → 147.1/101.1	5.5 ± 0.6
1-*O*-CGlc	2.96	360.3 → 163.3/145.3	8800 ± 200
FGlc (sum of 3 isomers)	26.97, 29.91, 30.76	374.4 → 177.2/145.3	28.6 ± 0.7
Arbutin	3.71	290.2 → 180.3/163.3	5700 ± 100

3/4-*O*-*p*-coumaroyl glucaric acid **GA2** with 2450 ppm in the dried leaf material showed the highest amount of SG among all glucaric acid conjugates. In total, 6 isomers of *p*-coumaroyl glucaric acid were detected with a sum of 6400 ppm. The isolated 3/4-*O*-isomer, with an estimated percentage of about 40%, was the major structure. Same resulted for 3/4-*O*-caffeoyl glucaric acid **GA1** with 1250 ppm representing about 60% of 4 isomers totaling about 2010 ppm. The feruloyl glucaric acids were estimated with about 2200 ppm in total, while the most abundant isomer had 625 ppm (30% of the total). Compared with each other, *p*-coumaroyl glucaric acids had 3 times higher concentrations in SG compared to the caffeoyl and feruloyl derivatives. In phytochemical literature, almost only quantitative results for caffeoyl glucaric acids were reported. In leaves of *Solanum esculentum* (tomato) concentrations between 661 and 1850 ppm were determined for the dominant isomer (structure assignment solely based on HRMS data) depending on various varieties, which is very comparable to our findings for SG.^23^ In contrast, feruloyl and *p*-coumaroyl glucaric acids were almost absent in tomatoes with just 10–50 ppm each.^23^ Analyses of calafate berries also showed similar results for caffeoyl glucaric acids. Ruiz et al. determined 439 – 2340 ppm in different berry samples while the isolated 3- or 4-*O*-isomers had the highest amount.^33^ Taken together, the high concentrations of *p*-coumaroyl but also feruloyl glucaric acids, we found herein for SG are unique and never have been published for other plants.

### Identification and Quantitation of Quinic Acid Derivatives

Commercially available reference material for 3-*O*- and 5-*O*-caffeoyl-, feruloyl-, and *p*-coumaroyl quinic acid was used to develop an LC/MS-MRM quantitation method. All 6 structures were then unequivocally identified in the crude leaf extract of SG by retention time and fragmentation (Figure 7B). As seen from Tables 4, 3-*O*-cinnamic quinic acids dominated over 5-*O*-derivatives by a factor of 2 to 5. 3-*O*-Caffeoyl quinic acid had a concentration of 560 ppm with respect to dried leaf material. This value is in the same range as published for other *Solanaceae* such as *Cestrum poeppigii* (216–595 ppm), *Solanum tuberosum* (318–1625 ppm), or *Nicotiana tabacum* L. (332 ppm).^37−39^ Almost the same amount was found for 3-*O*-feruloyl quinic acid, with 580 ppm, while a lower amount of 355 ppm was determined for 3-*O*-*p*-coumaroyl quinic acid. Comparable contents of 3-*O*-*p*-coumaroyl quinic acid were determined in *Hemerocallis citrina* Baroni ranging from 185 to 740 ppm within 13 different sample batches.^40^ In coffee (*Coffea* spp.) up to 2180 ppm were determined by Ortiz et al.^41^ Few data were published for feruloyl quinic acids in the literature. Only low amounts, between 15 and 26 ppm, were reported for tomato and potato leaves. Thus, SG had up to 20 times higher levels in comparison to other *Solanaceae*.^16,18^ The in principle lower amounts of 5-*O*- compared to 3-*O*-isomers were consistent to publications on potato cultivars for 5-*O*-caffeoyl- and 5-*O*-*p*-coumaroyl quinic acids and have been validated by the biosynthesis.^38,40,42^ In total, about 2000 ppm of quinic acids were found in SG dried leaf material, which was clearly below the level of obtained glucaric acids with 10600 ppm. This was also demonstrated for tomatoes before, where the sum of 4 caffeoyl quinic acid isomers resulted in 560 ppm, while 2 isomers of caffeoyl glucaric acid were determined at 180–532 and 661–1850 ppm, respectively.^16,23^ This reflects a comparable ratio of about 1 to 5, as observed during the present analyses in SG.

### Synthesis, Identification, and Quantitation of Cinnamic Acid Glucosides

Mass spectrometric evaluation of the material from the polar prefractionation step not only led to above glucaric acids but also to the identification of cinnamic acid glucosides, e.g., pseudo-molecular ion patterns of 360.4 *m*/*z* for [M+NH_4_]^+^, 365.3 *m*/*z* for [M + Na]^+^, and 707.4 *m*/*z* for [2M+Na]^+^, respectively. The molecular ion [M+NH_4_]^+^ led to characteristic ions for caffeic acid in MS/MS fragmentation (*m*/*z*: 325.3, 163.3, 145.3, and 116.9).^23^ The complementary part was detected with fragment ions of 180.4 as [M + H]^+^ and 203.4 as [M + Na]^+^ indicating a hexose (glucose) unit. Due to the lack of commercial reference material for confirmation, first the 3-*O*-caffeoyl and 3-*O*-feruloyl glucosides were independently synthesized following a synthesis route published by Jaiswal et al.^21,22^ The hydroxyl groups of cinnamic acid were protected via allylation. Then, the carboxylic group was activated as an acid chloride and coupled to 1,2:5,6-di-isopropylidene-α-d-glucofuranose. Finally, the protecting groups were eliminated in two steps to obtain mixtures of 3-*O*-caffeoyl- and 3-*O*-feruloyl-(*α/β*)-glucosides.^21,22^ This strategy was then transferred to the synthesis of 1-*O*-caffeoyl-β-glucoside starting from 2,3,4,6-tetra-*O*-benzyl-β-glucopyranose. All intermediates and final products were verified via HRMS and NMR spectroscopy and were identical to literature;^22^ however, data for 1-*O*-caffeoyl-glucoside were assessed from isomeric mixtures. Based on our synthesis, specific spectral information for the β-isomer is now given in the Materials and methods section and in Figure S7.

Again, the synthesized reference materials were used to establish an LC/MS-MRM method for the quantitation of caffeoyl and feruloyl glucosides, respectively. This method was also used to simulate MRM transitions for *p*-coumaroyl glucosides based on our experiments and the literature.^19^ The 3-*O*-caffeoyl glucosides gave a *t*_R_ = 6.52 min and *t*_R_ = 8.21 min for the α- and β-isomer, respectively, and the 3-*O*-feruloyl glucosides at *t*_R_ = 16.73 and 19.79 min. However, none of these 3-O-isomeres were identified in the SG extract (Figure 7C). Instead, the prominent peak at *t*_R_ = 2.82 min was identified as the 1-*O*-caffeoyl-β-glucoside by retention time and virtually the same fragmentation pattern as the synthesized authentic reference. Indeed, 1-*O*-caffeoyl glucoside was also determined as the main isomer found in various berry fruits, with maximum concentrations of 105 and 158 ppm in gooseberry and lingonberry, respectively.^19^ Another study revealed 6-*O*-caffeoyl glucoside as the quantitatively most important isomer with up to 390 ppm in tomato-based products.^43^ In SG, 1-*O*-caffeoyl glucoside had the highest amount at 8800 ppm in dry leaf material. This was almost twice as high as the content of arbutin, the published *p*-hydroquinone glucoside found in SG (5700 ppm). As the other signals for *p*-coumaroyl- and feruloyl glucosides had similar fragmentation patterns to the reference material (3-*O*-derivative) it can be anticipated that these peaks also belong to other 1/2/4/5-*O*- as well as cis/trans or α-/β-glucosides. In this case, the simulated MRM transitions can be used to estimate feruloyl glucosides at concentrations of about 29 ppm and *p*-coumaroyl glucosides at 5.5 ppm. Feruloyl- and *p*-coumaroyl glucosides were almost solely described qualitatively in the literature; thus, a comparison of our quantitative results is almost impossible. Obviously, syntheses of more authentic material are necessary to address this point.

In conclusion, the present investigation comprehensively extends the knowledge on phenolic compounds in *Solanum glaucophyllum* Desf., with 33 phenolic structures identified and quantified, whereby 27 of them were described for the first time in SG. Taken together, these secondary plant metabolites explain about 7.0% of the total dry matter. Quercetin glycosides are the major flavon-3-ol structures with 4.2% followed by 2.2% cinnamoyl derivatives (1.5% glucosides, 1.1% glucaric acids, and 0.2% quinic acids) and 0.6% arbutin. The verification of quercetin-7-*O*-β-glucosyl-3-*O*-β-(2’’-*O*-α-apiosyl)-rutinoside (glaucophylloside) must be emphasized due to the first description in the literature. Isolation and workup procedures will now be used to further unravel unknown 1,25(OH)_2_D_3_ glycosides to explain the high concentrations of free 1,25(OH)_2_D_3_ levels that can be observed after enzymatic hydrolyses to understand the physiological impact of SG on animals in more detail.

## Supporting Information Available

Figure S1: NMR spectra of 7-*O*-β-glucosyl-rutin **4.** Figure S2: NMR spectra of 7-*O*-β-glucosyl-α-apiosyl-rutin **5.** Figure S3: NMR spectra of 3/4-*O*-caffeoyl glucaric acid. Figure S4: NMR spectra of 3/4-*p*-*O*-coumaroyl glucaric acid. Figure S5: NMR spectra of 3-*O*-caffeoyl glucoside. Figure S6: NMR spectra of 3-*O*-caffeoyl glucoside. Figure S7: NMR spectra of 1-*O*-caffeoyl-β-glucoside. Figure S8: PMAA of 7-*O*-glucosyl-rutinoside. Table S1: MRM data for quantitation of cinnamic acid derivatives and arbutin. Table S2: validation data for quantitation methods (PDF)
