# Supplementary material for: Comprehensive Analysis of Phenolic Compounds in Solanum glaucophyllum Desf
Source: J Agric Food Chem. 2025 Mar 19;73(13):7741–54. doi: 10.1021/acs.jafc.4c11264 (PMC11969645; doi:10.1021/acs.jafc.4c11264)
Supplement: Supplementary file 1 — jf4c11264_si_001.pdf [file jf4c11264_si_001.pdf]

SUPPORTING INFORMATION

Comprehensive Analysis of Phenolic Compounds in Solanum Glaucophyllum Desf.

Thomas Heymann[1], Sabrina Autzen[2], Marcus A. Glomb[1]

[1] Martin-Luther-University Halle-Wittenberg, Institute of Chemistry – Food Chemistry, Kurt-Mothes-Str. 2, D-06120 Halle/Saale, Germany

[2] Herbonis Animal Health GmbH, Rheinstrasse 30, CH-4302 Augst BL, Switzerland

-Figure S1: NMR spectra 7-*O*-β-glucosyl-rutin 4

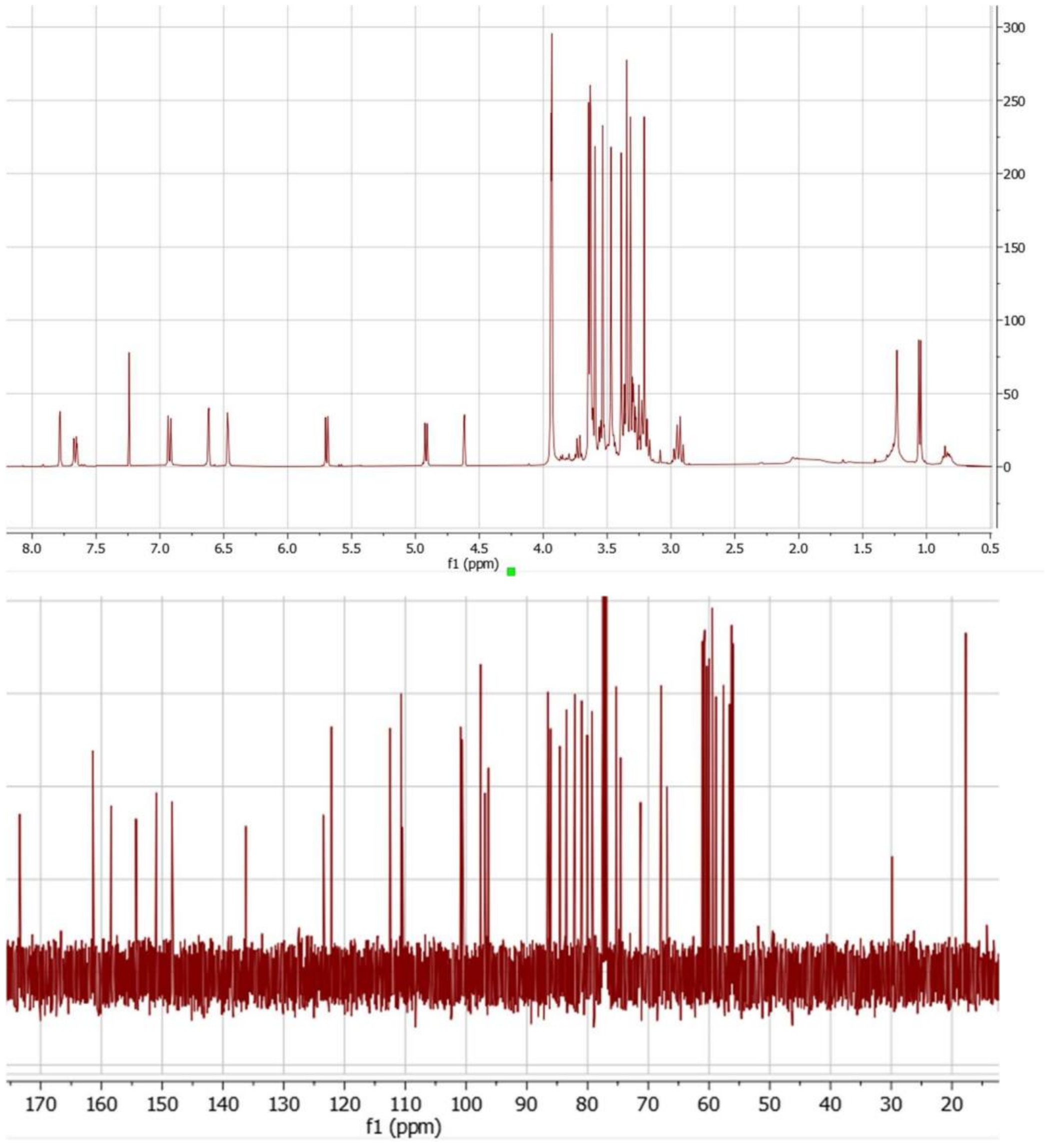

^{1}H-NMR (top) and ^{13}C-NMR (bottom)

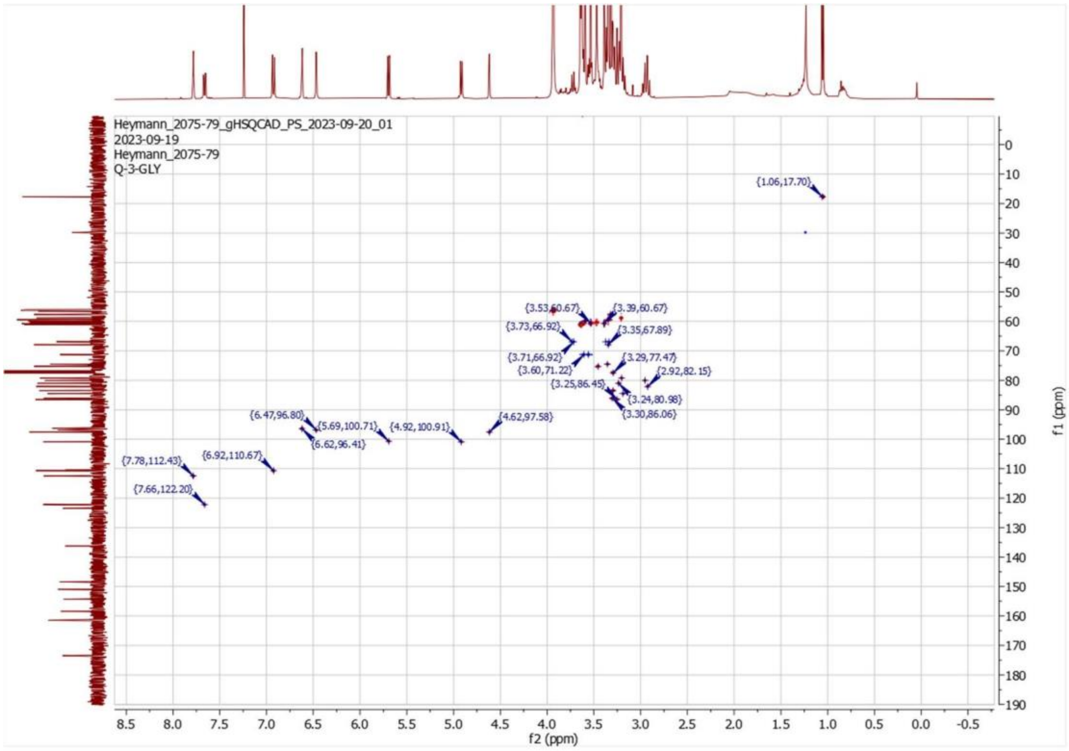

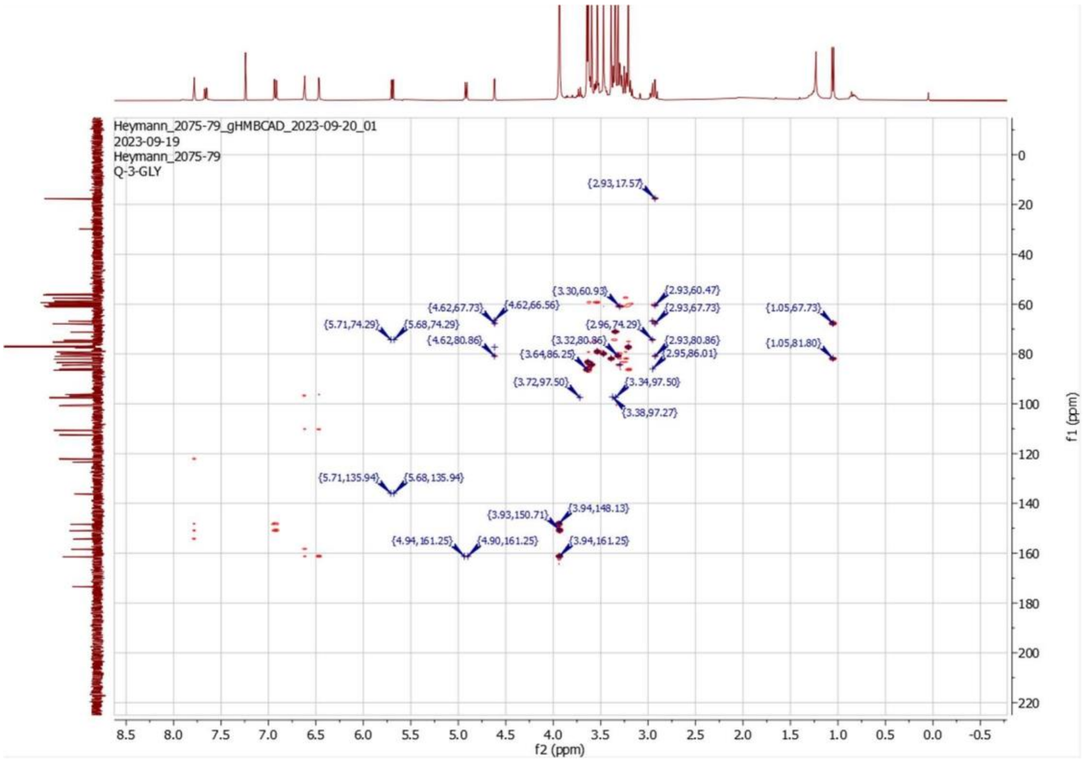

HSQC-NMR (top) and HMBC-NMR (bottom)

-Figure S2: NMR spectra 7-*O*-β-glucosyl-α-apiosyl-rutin 5

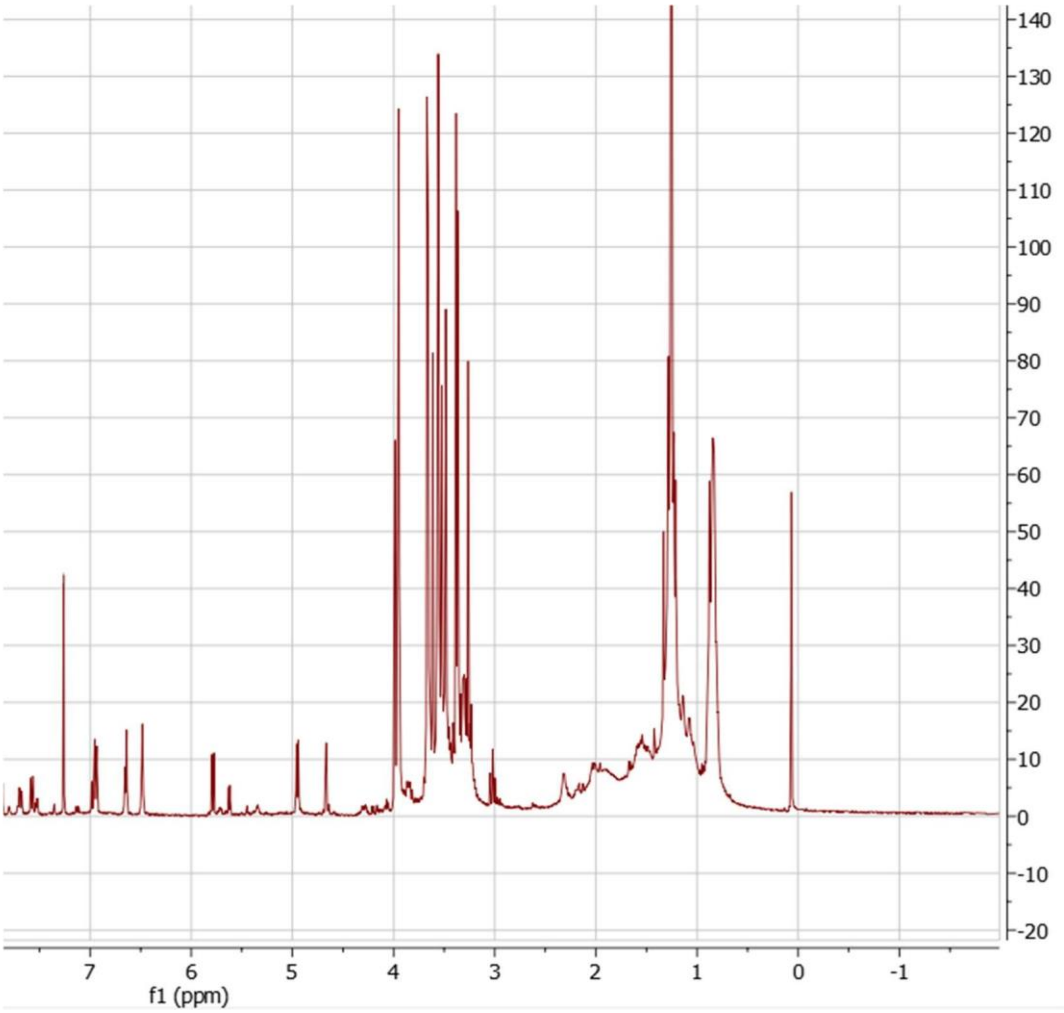

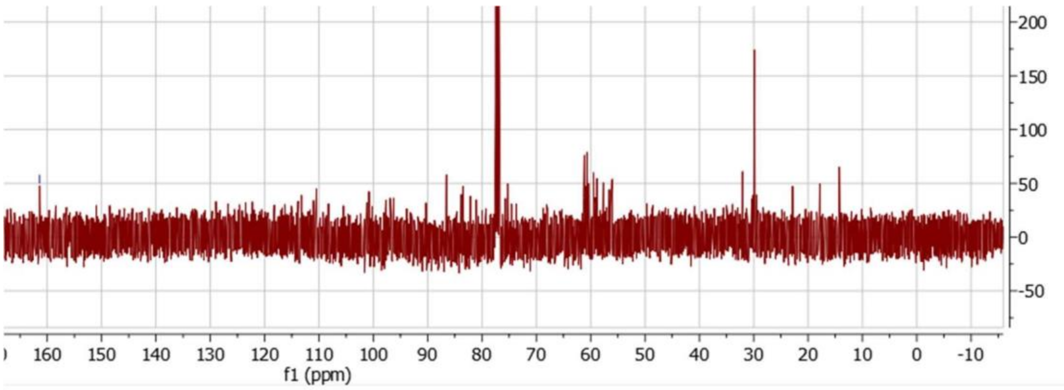

^{1}H-NMR (top) and ^{13}C-NMR (bottom)

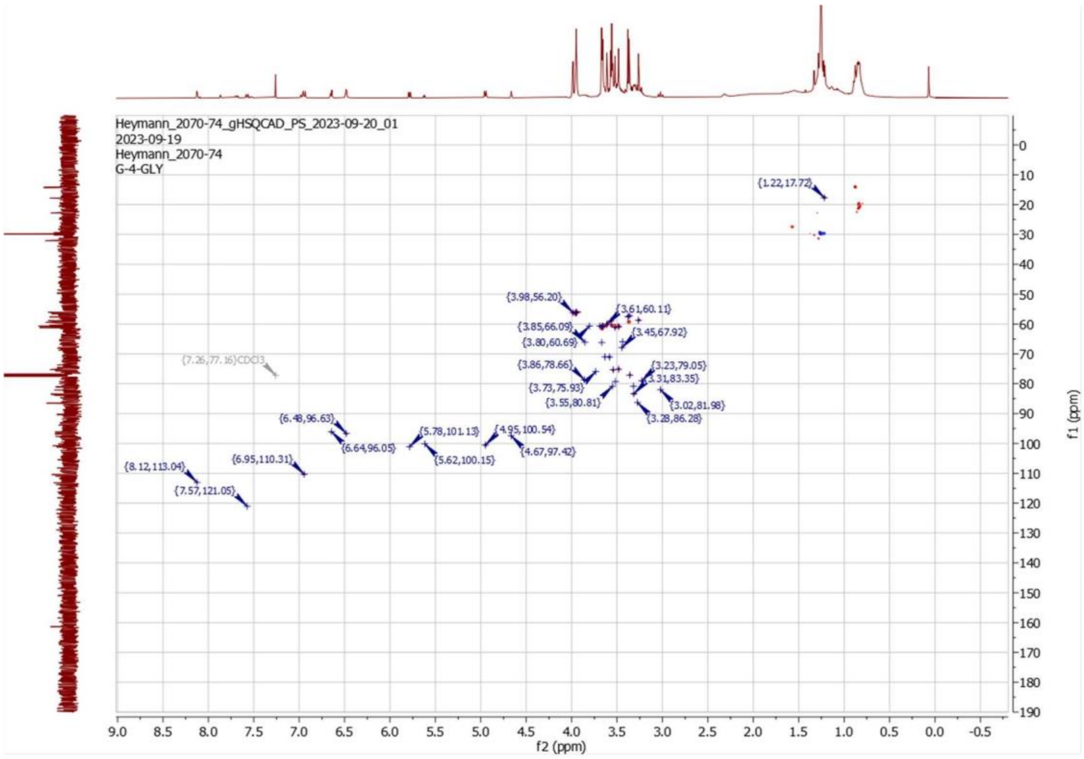

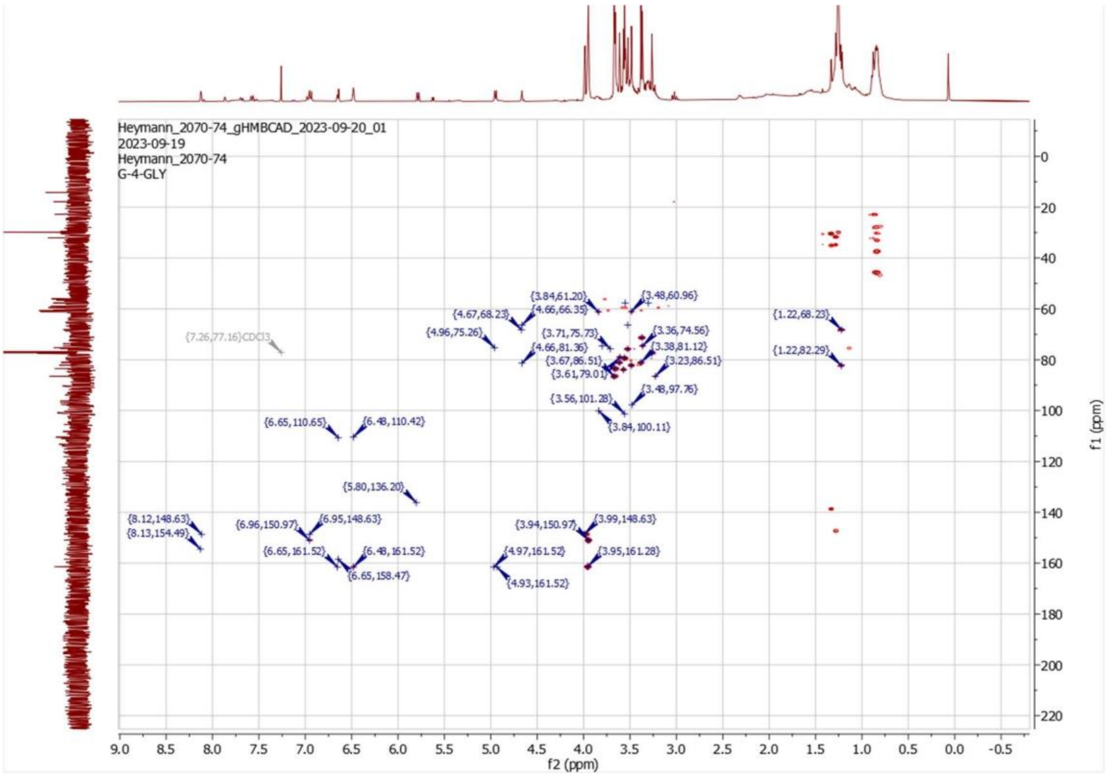

HSQC-NMR (top) and HMBC-NMR (bottom)

-Figure S3: NMR spectra 3/4-O-caffeoyl glucaric acid GA1

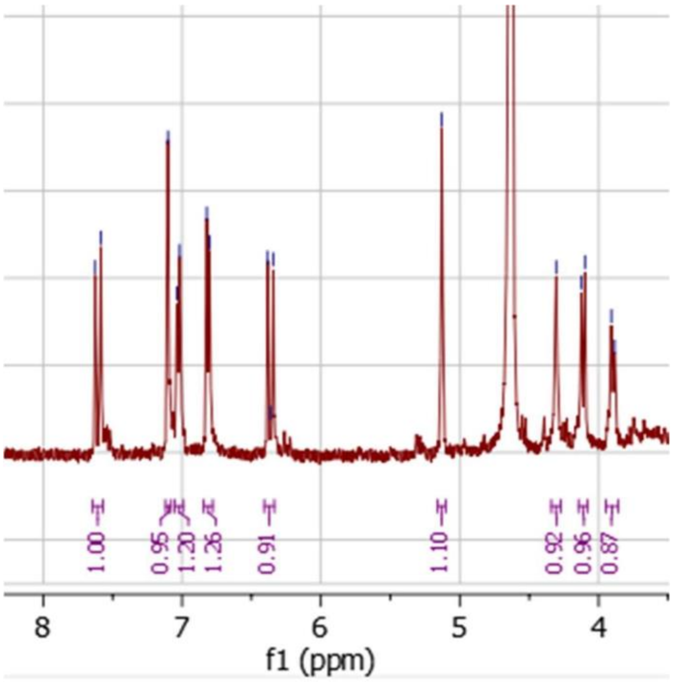

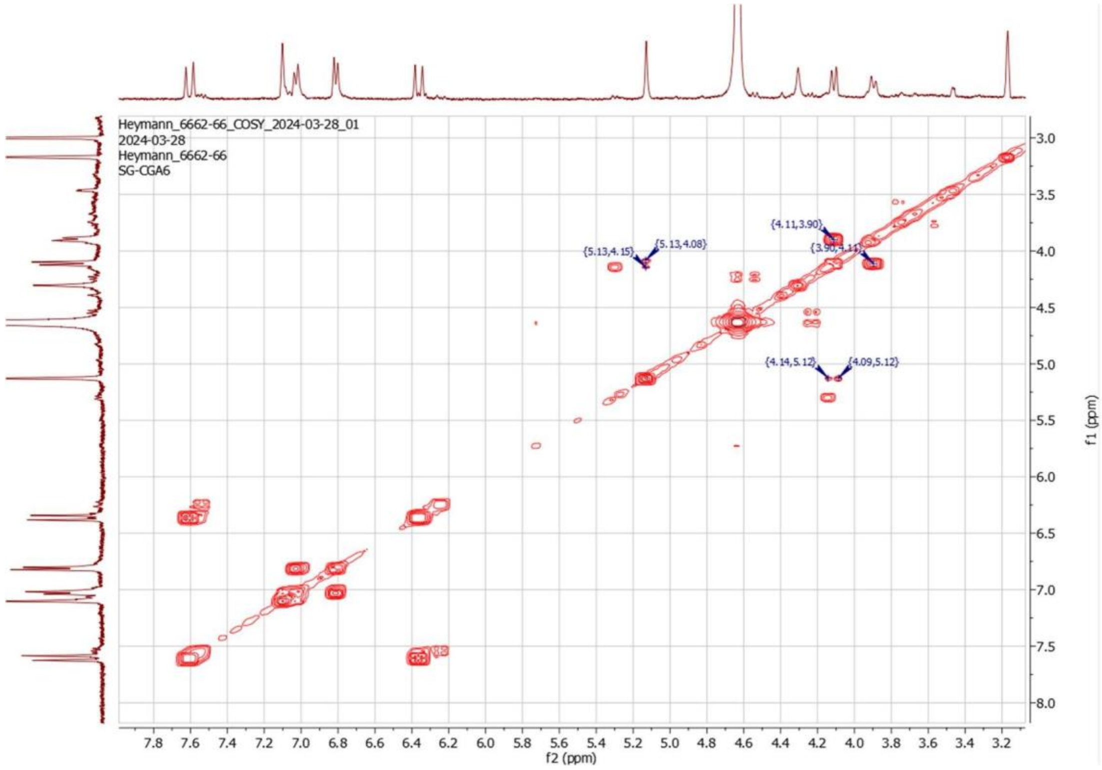

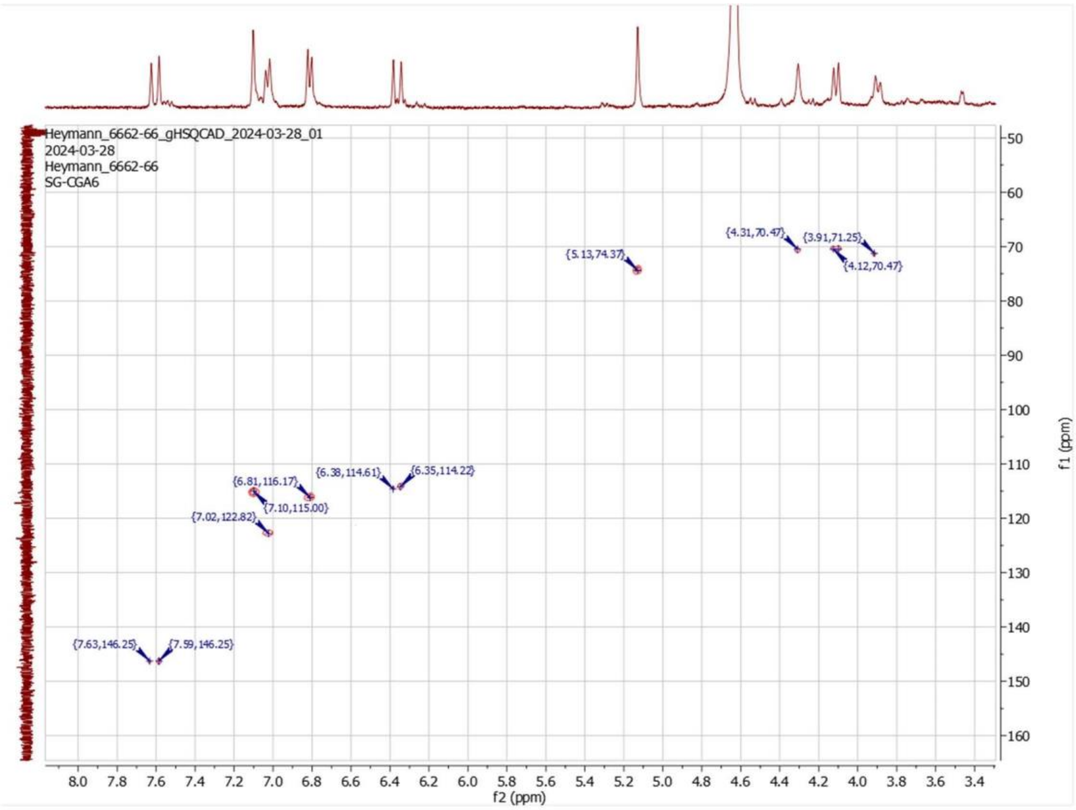

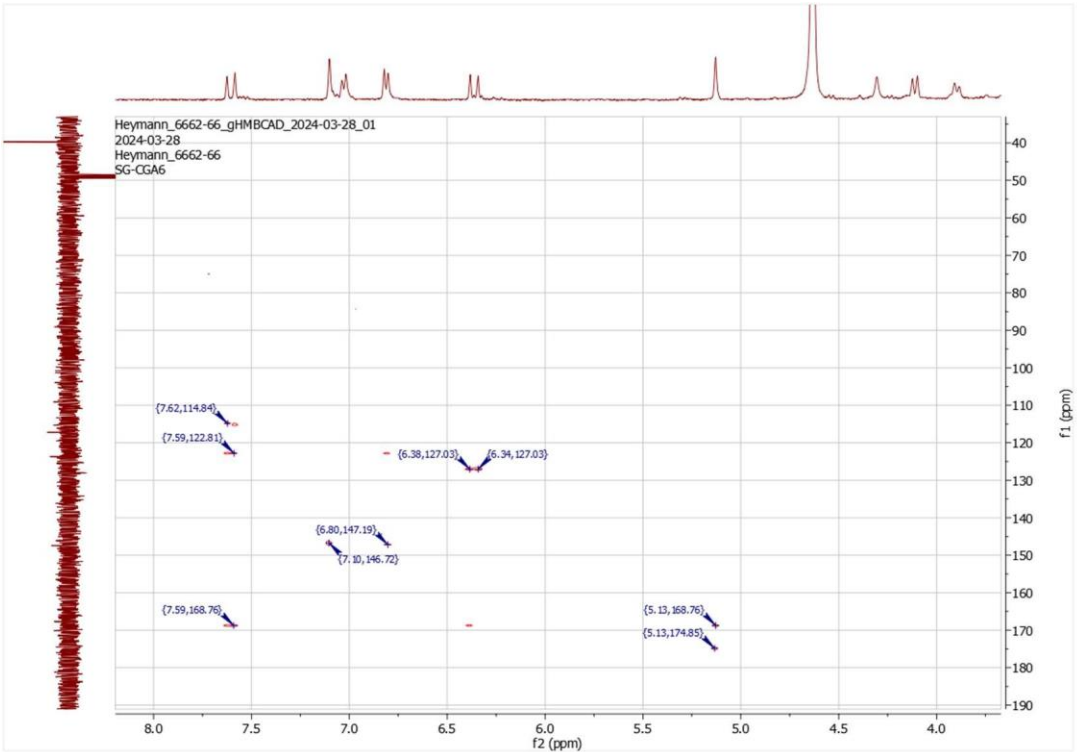

HSQC-NMR (top) and HMBC-NMR (bottom)

-Figure S4: NMR spectra 3/4-*p*-O-coumaroyl glucaric acid GA2

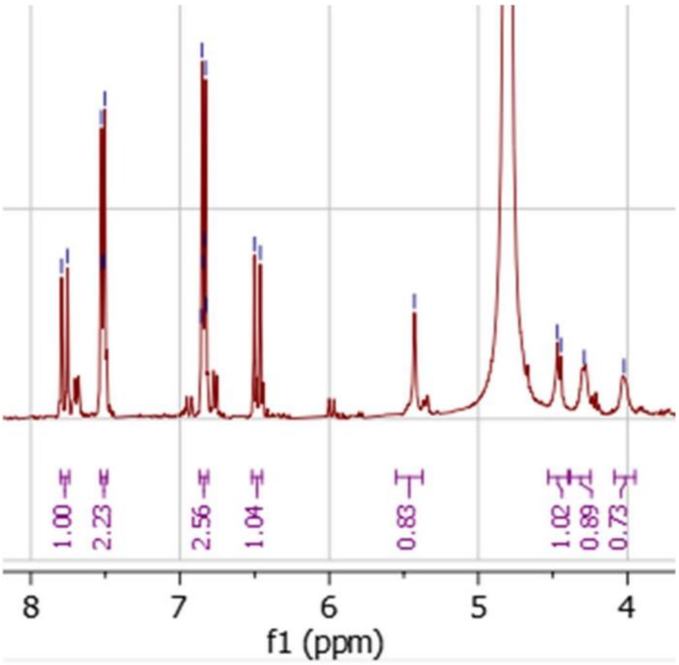

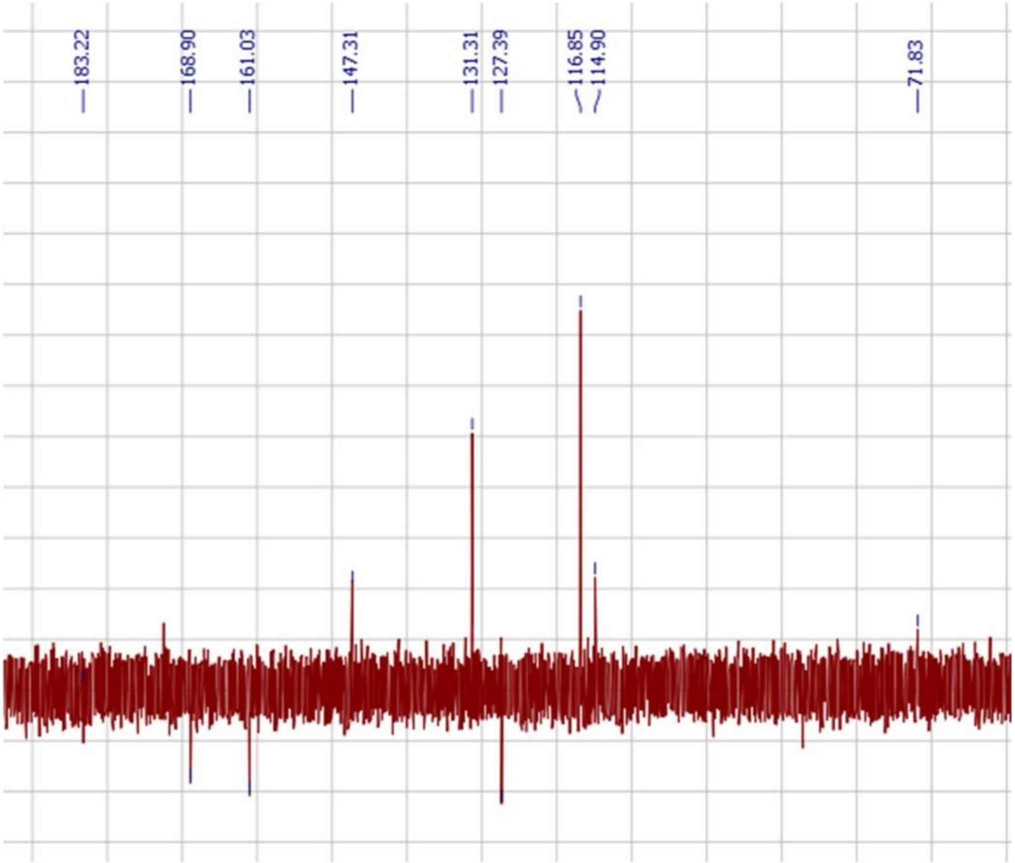

^{1}H-NMR (top) and ^{13}C-NMR (bottom)

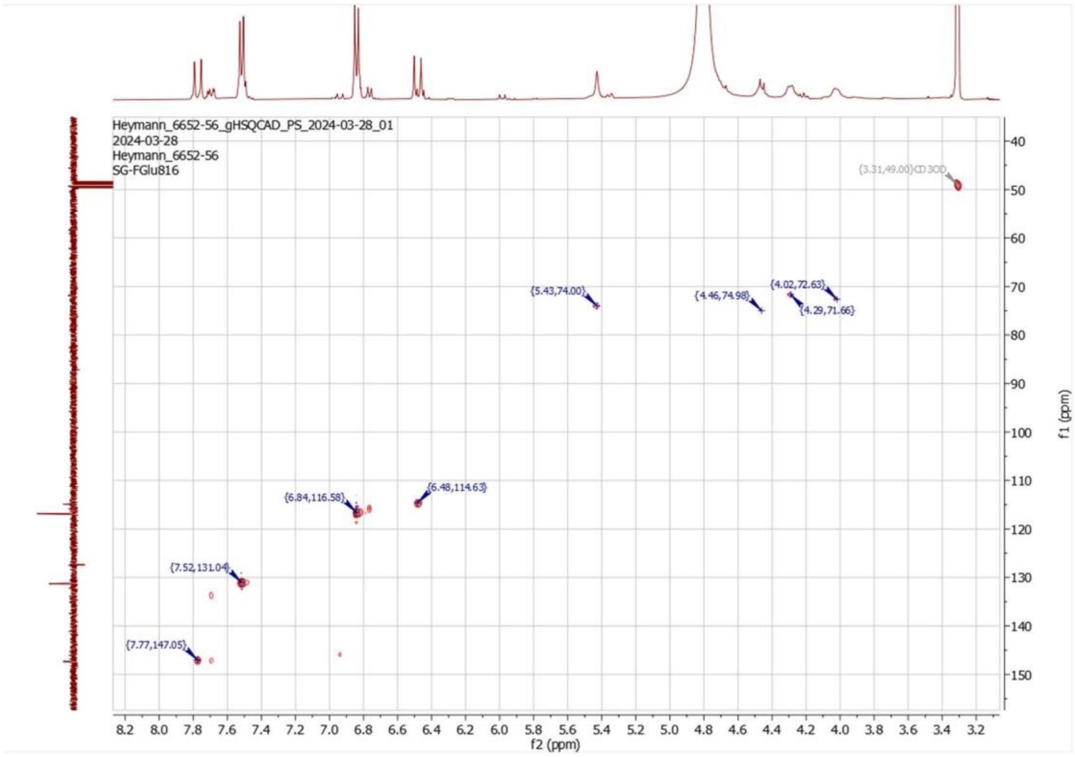

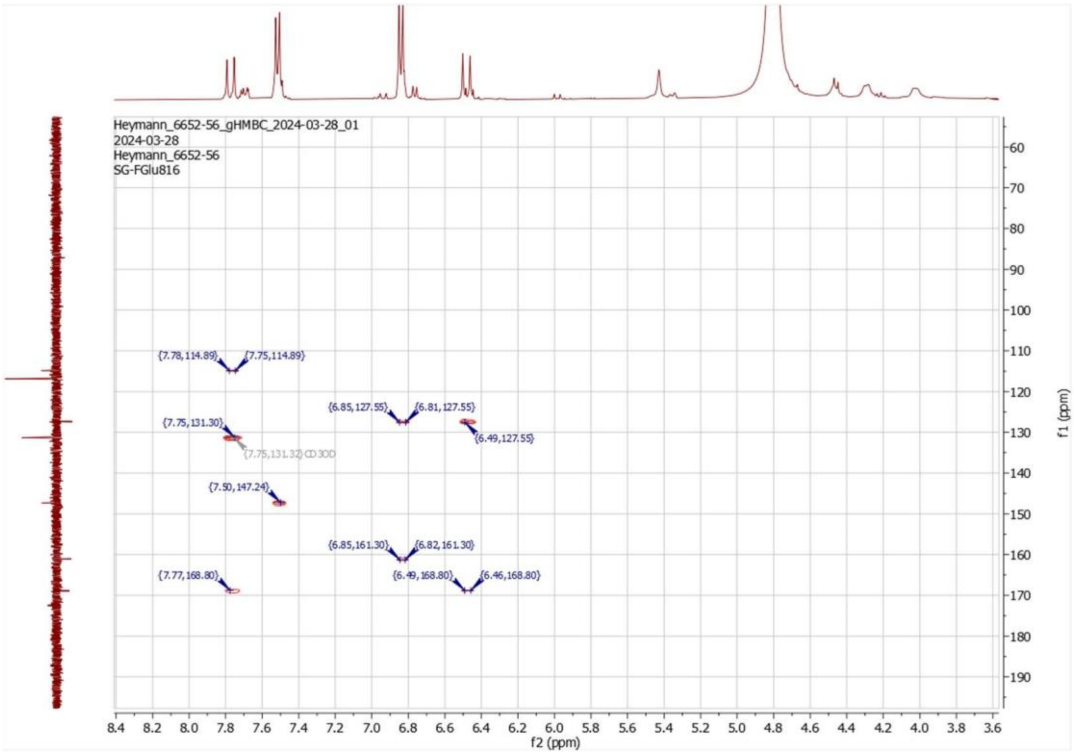

HSQC-NMR (top) and HMBC-NMR (bottom)

-Figure S5: NMR spectra 3-*O*-caffeoyl glucoside

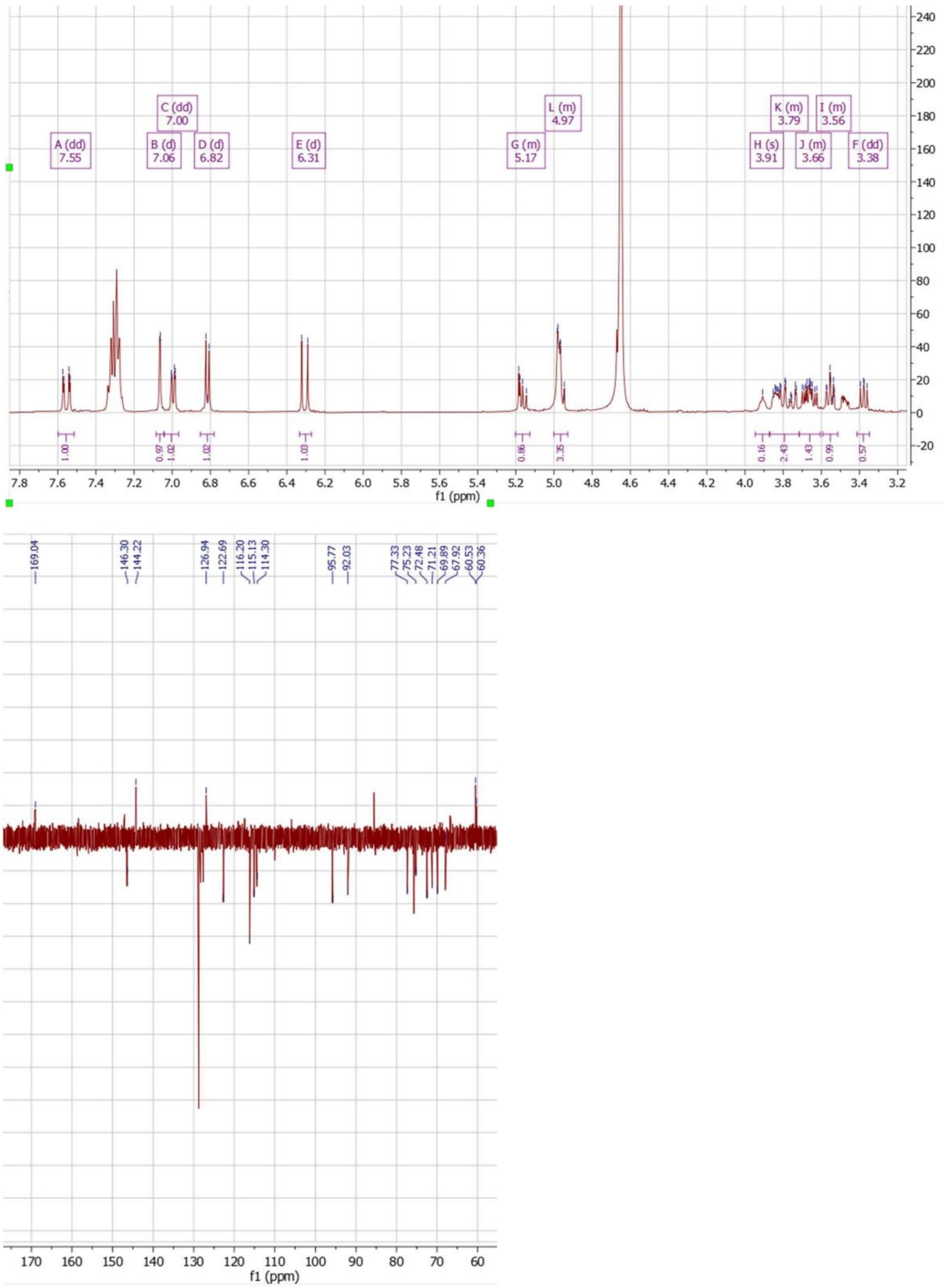

^{1}H-NMR (top) and ^{13}C-NMR (bottom)

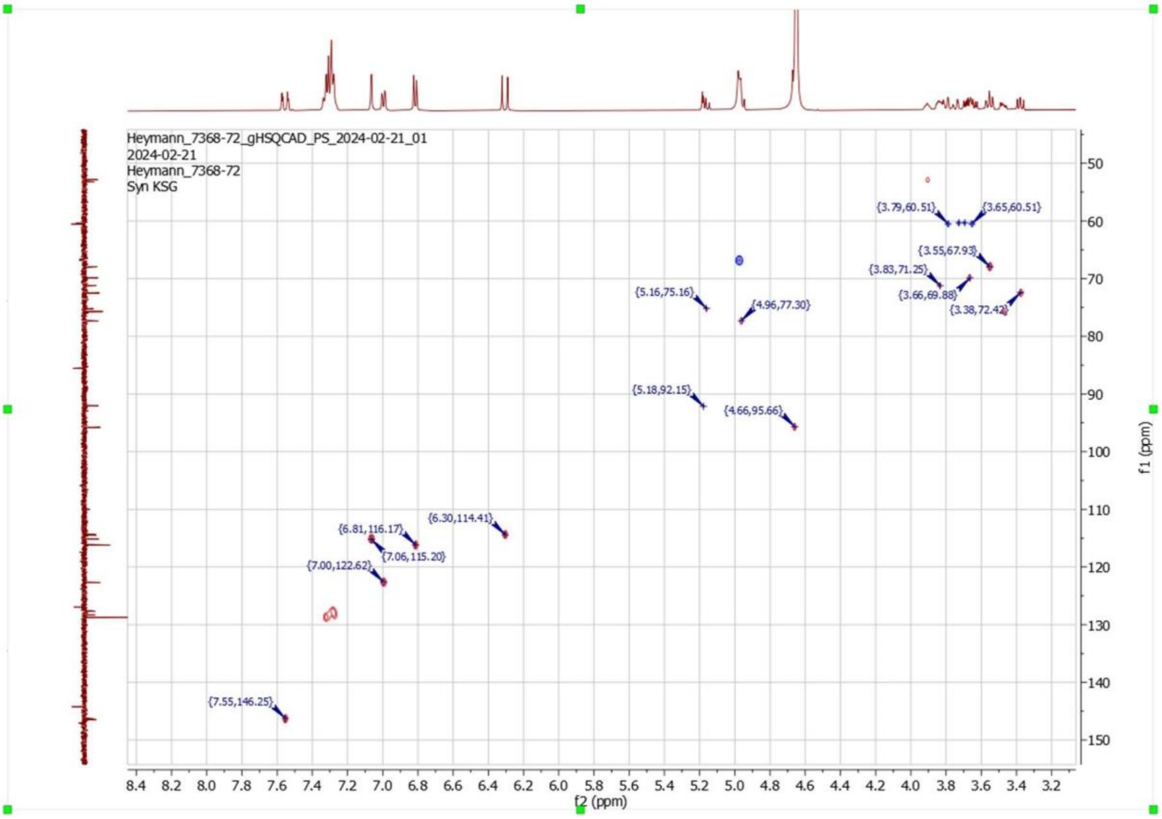

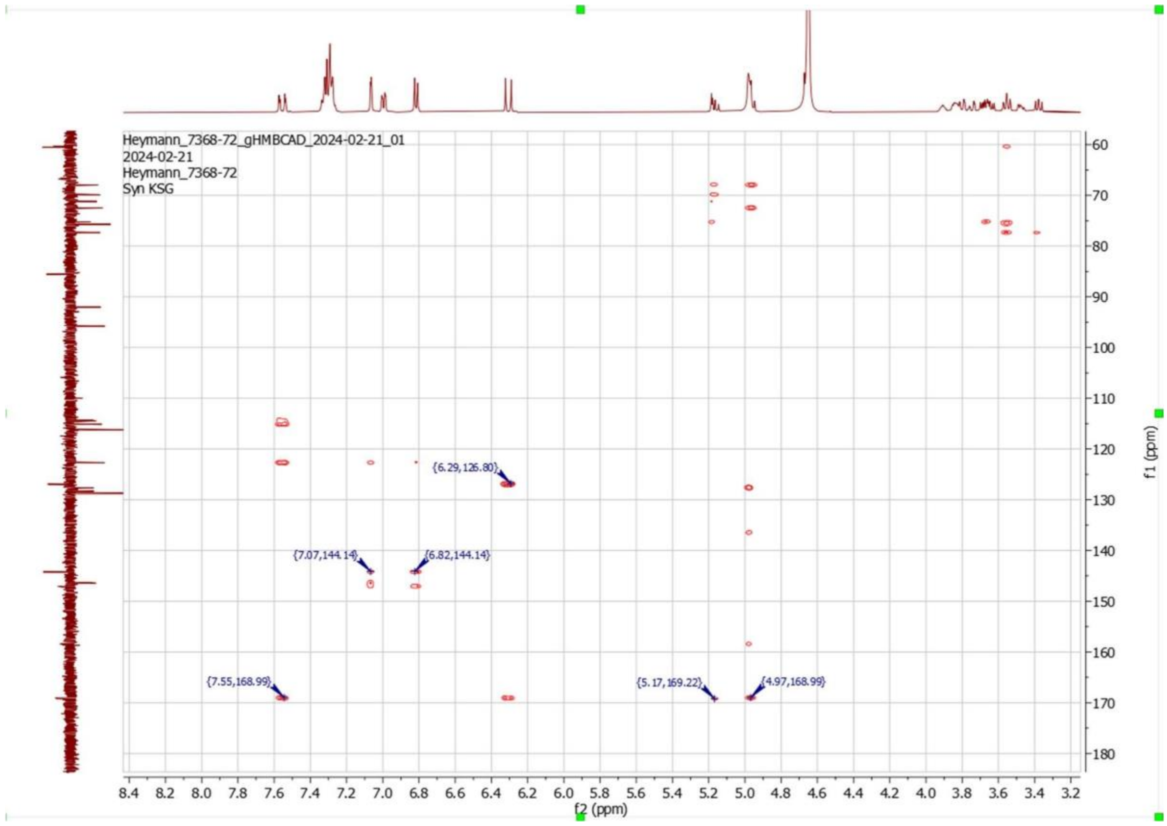

HSQC-NMR (top) and HMBC-NMR (bottom)

-Figure S6: NMR spectra 3-*O*-feruloyl glucoside

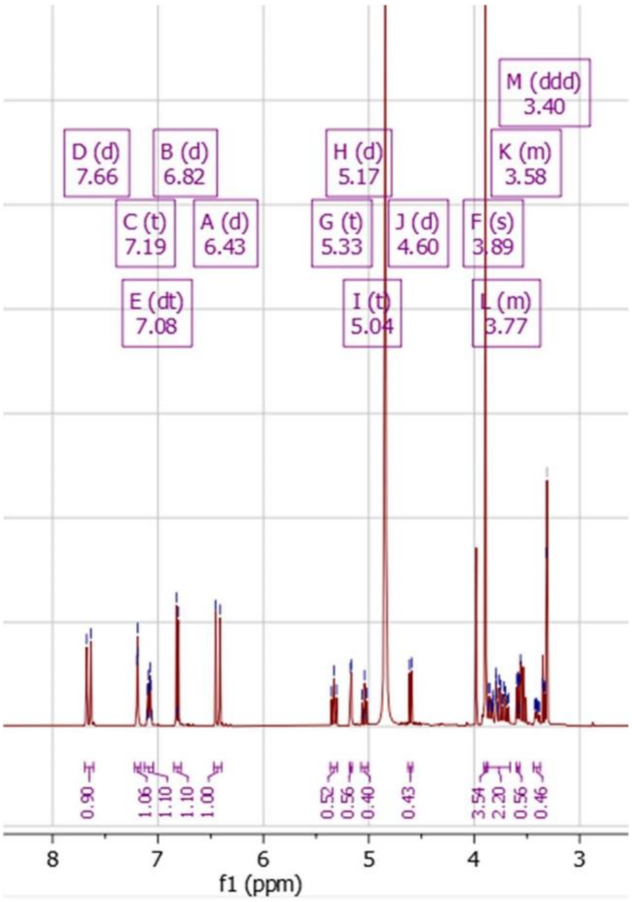

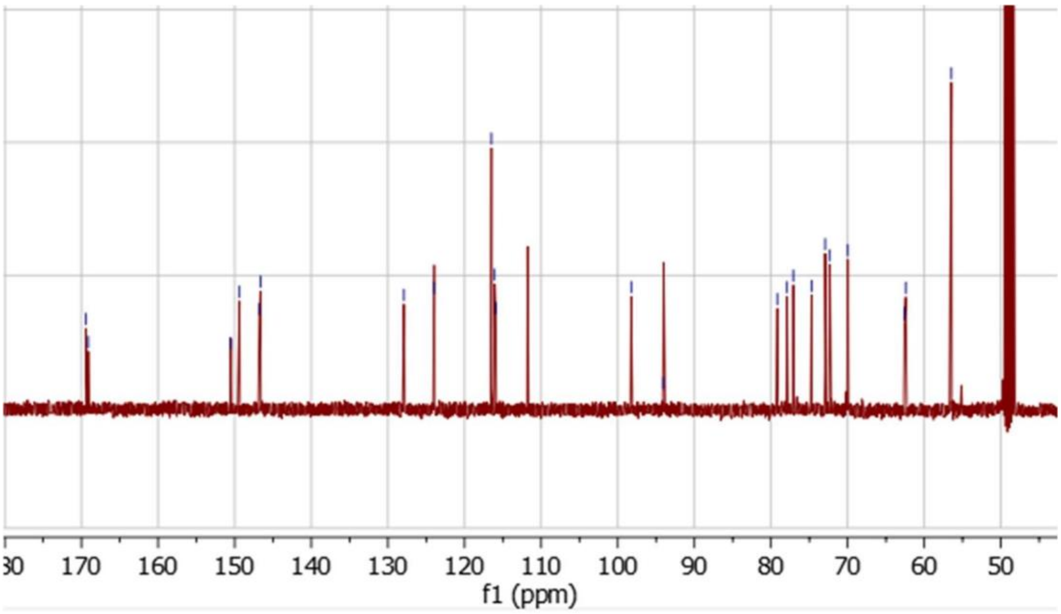

^{1}H-NMR (top) and ^{13}C-NMR (bottom)

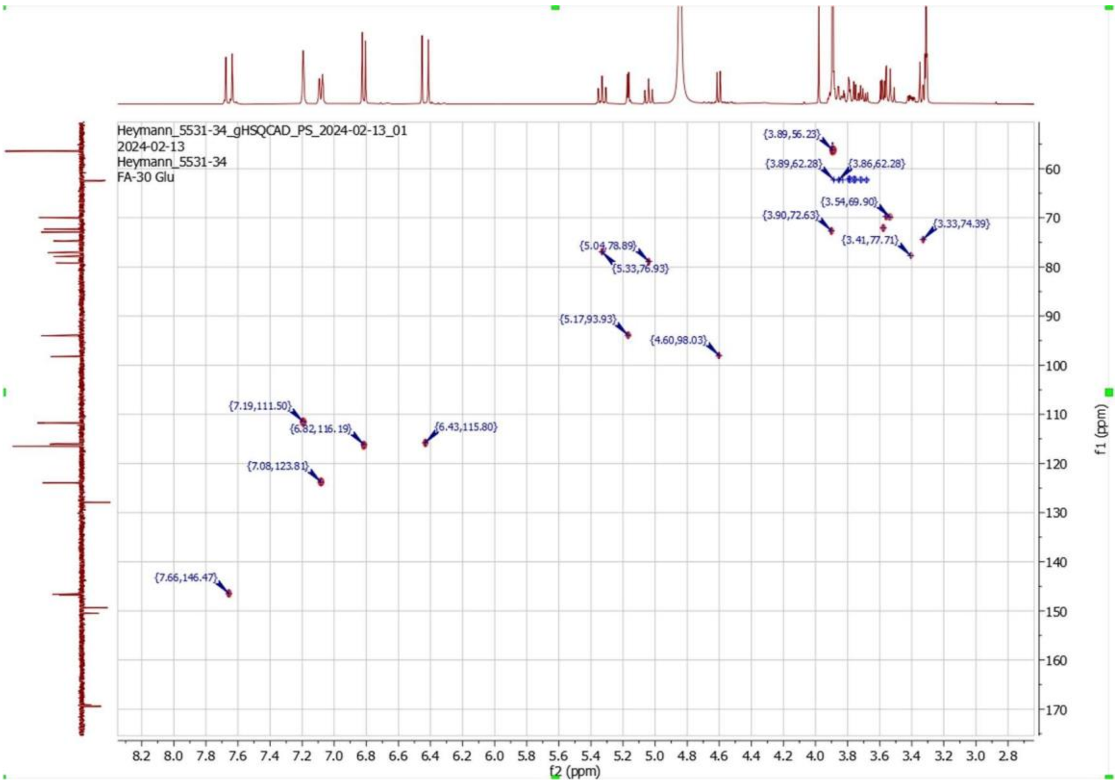

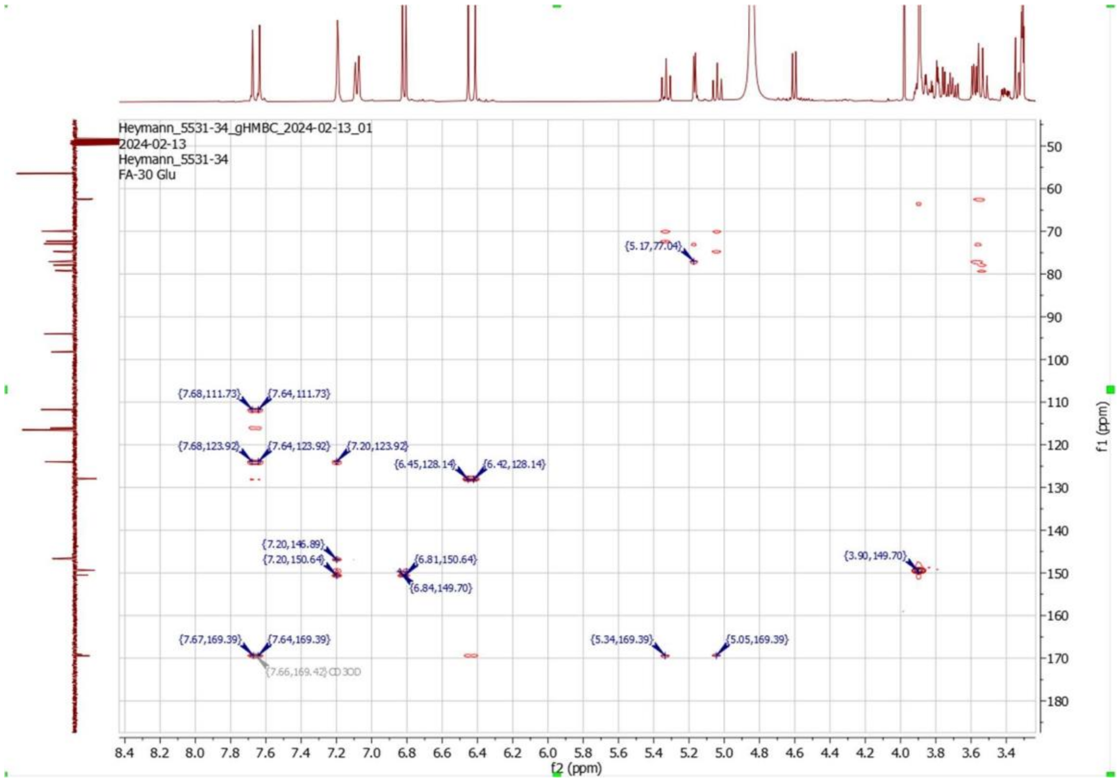

HSQC-NMR (top) and HMBC-NMR (bottom)

-Figure S7: NMR spectra 1-*O*-β-caffeoyl glucoside

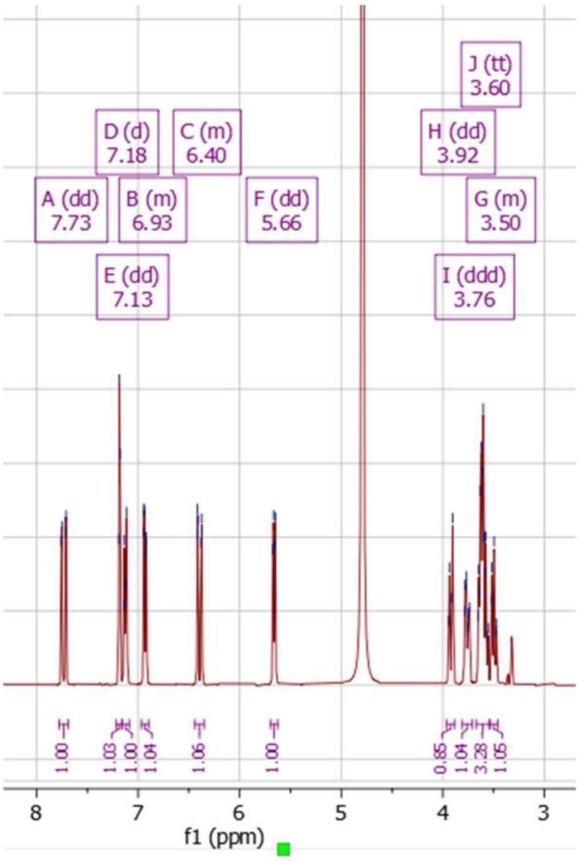

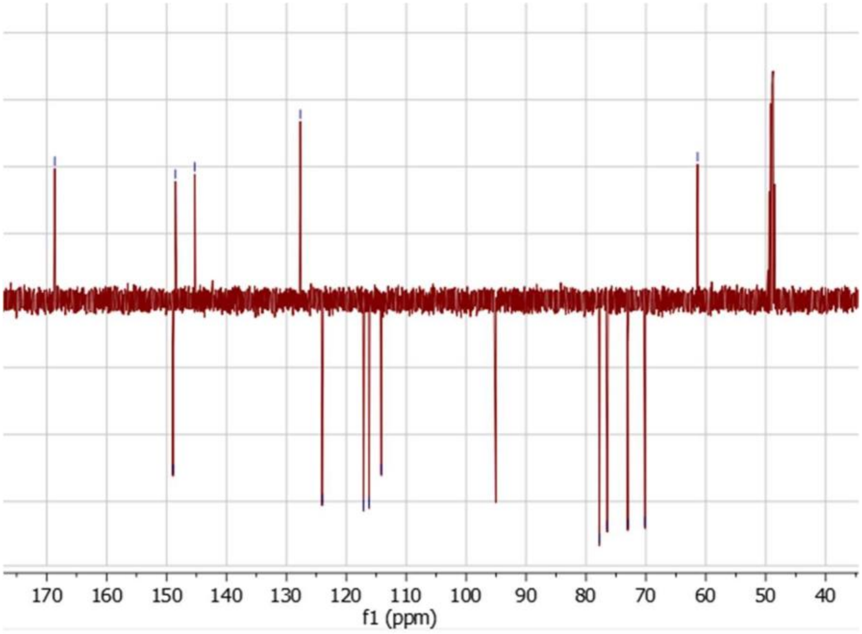

^{1}H-NMR (top) and ^{13}C-APT-NMR (bottom)

-Figure S8: PMAA of 7-*O*-β-Glucosyl-rutinoside

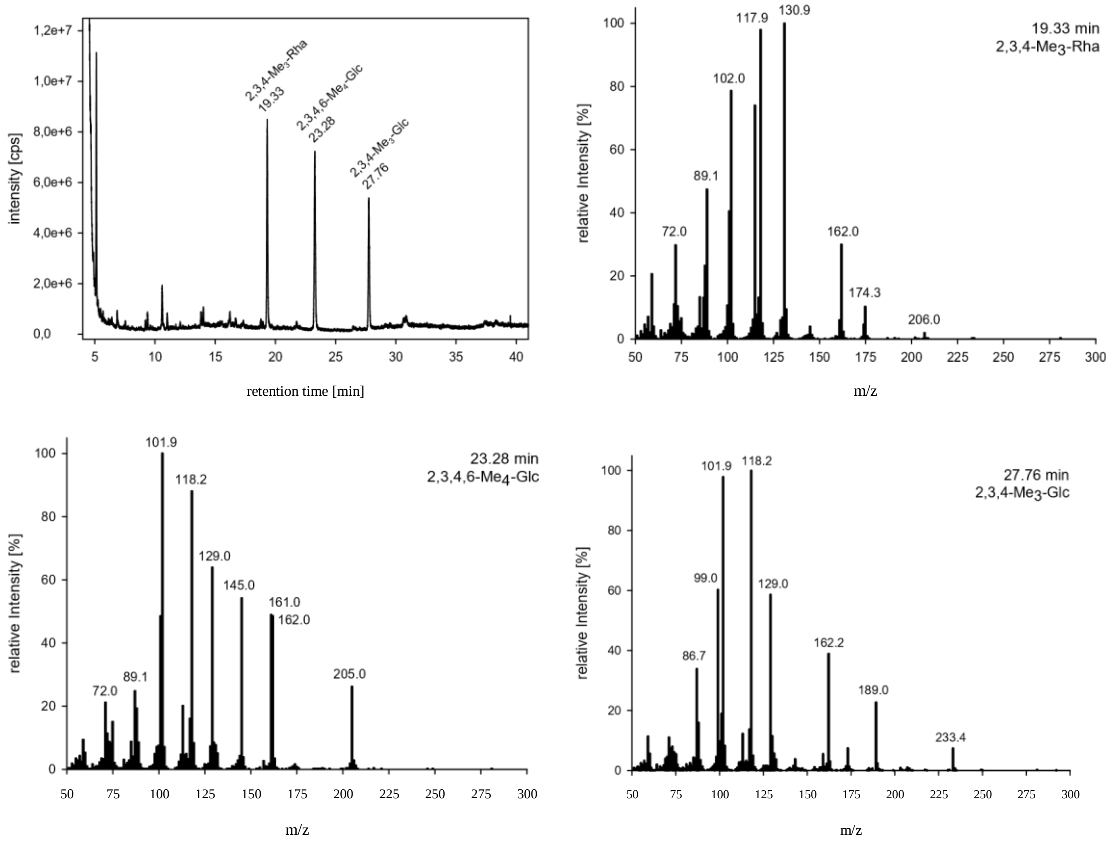

-Tab. S1: MRM data for quantitation of cinnamic acid derivatives and arbutin.

compound	MS1	MS2		DP	CE	CXP
5-O-p-coumaroylquinic acid	339.3	307.3	QL1	72.5	20.0	20.0
		293.2	QL2	72.5	20.0	20.0
		147.1	QN	72.5	23.5	30.0
3-O-p-coumaroylquinic acid	339.3	307.3	QL1	40.8	14.5	19.0
		293.2	QL2	40.8	16.0	16.0
		147.1	QN	40.8	17.0	40.0
5-O-caffeoylquinic acid	355.4	323.2	QN2	72.5	20.0	20.0
		309.4	QL	72.5	20.0	20.0
		163.0	QN1	72.5	23.5	30.0
3-O-caffeoylquinic acid	355.4	323.5	QN2	40.8	14.5	19.0
		309.4	QL	40.8	16.0	16.0
		163.0	QN1	40.8	17.0	40.0
5-O-feruloylquinic acid	369.3	337.3	QN2	72.5	20.0	20.0
		323.4	QL	72.5	20.0	20.0
		177.2	QN1	72.5	23.5	30.0
3-O-feruloylquinic acid	369.3	337.3	QL1	40.8	14.5	19.0
		323.4	QL2	40.8	16.0	16.0
		177.2	QN	40.8	17.0	40.0
3/4-O-p-coumaroylglucaric acid	374.2*	339.3	QL2	51.1	11.0	12.9
		147.3	QN	51.1	22.2	25.0
		119.3	QL1	51.1	54.5	10.8
3/4-O-caffeoylglucaric acid	390.2*	163.3	QN	59.9	21.9	10.8
		145.4	QL1	59.9	51.0	13.5
		117.3	QL2	59.9	64.0	8.8
feruloylglucaric acid	404.2*	194.1	QN	59.9	21.9	10.8
		177.2	QL1	40.0	30.0	15.0
		145.2	QL2	40.0	30.0	15.0
p-coumaroylglucoside	344.3*	147.1	QN	52.0	17.0	28.5
		129.1	QL1	52.0	45.0	24.5
		101.1	QL2	52.0	57.0	18.9
1-O-caffeoylglucoside	360.3*	325.3	QL3	52.0	8.5	13.0
		163.3	QN	52.0	17.0	28.5
		145.3	QL1	52.0	45.0	24.5
		116.9	QL2	52.0	57.0	18.9
3-O-caffeoylglucoside	360.3*	325.3	QL3	52.0	8.5	13.0
		163.3	QN	52.0	17.0	28.5
		145.3	QL1	52.0	45.0	24.5
		116.9	QL2	52.0	57.0	18.9
3-O-feruloylglucoside	374.4*	339.4	QL3	45.0	10.0	15.5
		177.2	QN	45.0	21.5	12.5
		145.3	QL1	45.0	52.5	24.5
		117.1	QL2	45.0	60.0	17.1
arbutin	290.4*	180.4	QN	51.5	10.5	15.0
		163.3	QL1	51.5	14.0	13.1
		145.2	QL2	51.5	19.5	10.8
		85.0	QL3	51.5	30.0	15.7

* ammonia adduct

QN: quantifier – fragment ion used for quantitation, QL1 / QL2: qualifier – fragment ion for identification / verification of target molecule

-Tab. S2: Validation data for quantitation methods.

Parameter	Quinic Acids	Cinnamoyl Glucoside	Cinnamoyl Glucaric Acids	Arbutin	Quercetin Derivatives
method	LC-MS/MS	LC-MS/MS	LC-MS/MS	LC-MS/MS	HPLC-UV
	pos. mode	pos. mode	pos. mode	pos. mode	350 nm
Linearity					
- range	10 – 250 µM	5 – 50 µM	10 – 200 µM	50 – 50 µM	15 –875 µM
- sensitivity*	322100	603000	185500	141700	19584400
- coefficient R	0.9987	0.9988	0.9994	0.9989	0.9992
Method precision					
- RSD (n = 6)	2.9 % (10 µM)	2.8 % (25 µM)	4.0 % (65 µM)	1.8 % (40 µM)	1.5 % (550 µM)
	1.8 % (30 µM)		4.6 % (20 µM)		1.4 % (270 µM)
					2.0 % (30 µM)
LOD	0.4 µM	0.5 µM	0.3 µM	0.03 µM	1.7 µM
LOQ	1.2 µM	1.5 µM	1.0 µM	0.08 µM	5.0 µM

* calculated by slope of calibration curve = cps * μM^{-1}, LOD = limit of detection determined by signal to noise ratio S/N = 3, LOQ = limit of detection quantitation by signal to noise ratio S/N = 10.
